# Supplementary material for: Linkages between maternal experience of intimate partner violence and child nutrition outcomes: A rapid evidence assessment
Source: PLoS One. 2024 Mar 18;19(3):e0298364. doi: 10.1371/journal.pone.0298364 (PMC10947923; doi:10.1371/journal.pone.0298364)
Supplement: S1 Appendix — (DOCX) [file pone.0298364.s001.docx]

**Supporting Information 1: Search Domains and Terms for All Databases**

Database(s): **Embase Classic+Embase**

- Dates searched:
  - 1947 to 2021 July 23, n=3285
  - 2021-2022 November 3, n=754

Search Strategy:

| **#** | **Searches** |
| --- | --- |
| 1 | nutritional disorder/ |
| 2 | feeding disorder/ or malnutrition/ or nutritional deficiency/ or overnutrition/ |
| 3 | cachexia/ or fetal malnutrition/ or malabsorption/ or protein deficiency/ |
| 4 | nutritional deficiency/ or protein deficiency/ or vitamin deficiency/ |
| 5 | rickets/ |
| 6 | protein deficiency/ or kwashiorkor/ |
| 7 | protein calorie malnutrition/ |
| 8 | starvation/ or food deprivation/ |
| 9 | overnutrition/ or obesity/ |
| 10 | morbid obesity/ |
| 11 | childhood obesity/ |
| 12 | wasting syndrome/ or body weight disorder/ |
| 13 | stunting syndrome/ |
| 14 | bottle feeding/ or infant feeding/ |
| 15 | breast feeding/ |
| 16 | birth weight/ or high birth weight/ or low birth weight/ |
| 17 | macrosomia/ |
| 18 | low birth weight/ or very low birth weight/ |
| 19 | small for date infant/ or intrauterine growth retardation/ |
| 20 | extremely low birth weight/ |
| 21 | weaning/ |
| 22 | maternal nutrition/ |
| 23 | crown rump length/ or anthropometric parameters/ |
| 24 | baby food/ or breast milk/ |
| 25 | artificial milk/ or milk substitute/ |
| 26 | exp nutrition policy/ |
| 27 | dietary reference intake/ or dietary intake/ |
| 28 | child nutrition/ or nutrition/ |
| 29 | anemia/ or iron deficiency anemia/ |
| 30 | iron deficiency anemia/ or iron deficiency/ |
| 31 | growth disorder/ or failure to thrive/ or growth retardation/ or stunting/ |
| 32 | body weight change/ or body weight gain/ or body weight loss/ |
| 33 | gestational weight gain/ |
| 34 | exp emaciation/ |
| 35 | cachexia/ |
| 36 | obesity/ or adolescent obesity/ or childhood obesity/ or diabetic obesity/ or maternal obesity/ |
| 37 | healthy diet/ or diet/ |
| 38 | body mass/ or anthropometric parameters/ |
| 39 | body fat distribution/ or adipose tissue/ or body composition/ |
| 40 | pregnancy diabetes mellitus/ or maternal diabetes mellitus/ |
| 41 | underweight/ |
| 42 | cephalometry/ |
| 43 | body growth/ |
| 44 | body height/ |
| 45 | body weight/ or ideal body weight/ |
| 46 | adrenarche/ |
| 47 | menarche/ or amenorrhea/ |
| 48 | protein intake/ or dietary intake/ |
| 49 | egg protein/ |
| 50 | meat protein/ or animal protein/ |
| 51 | milk protein/ |
| 52 | hunger/ |
| 53 | food insecurity/ |
| 54 | food security/ |
| 55 | beverage/ or carbonated beverage/ or "fruit and vegetable juice"/ or soft drink/ or sweetened beverage/ or tea/ |
| 56 | dietary supplement/ |
| 57 | fast food/ |
| 58 | iodine/ |
| 59 | exp vitamin/ |
| 60 | vitamin d/ |
| 61 | caloric intake/ |
| 62 | caloric restriction/ or diet restriction/ |
| 63 | portion size/ or food intake/ or food quantity/ |
| 64 | nutritional status/ |
| 65 | eating disorder/ or anorexia nervosa/ or avoidant restrictive food intake disorder/ or binge eating disorder/ or bulimia/ or emotional eating/ or food addiction/ or food aversion/ or food refusal/ or orthorexia/ or purging disorder/ |
| 66 | (iron deficien* or anemi* or anaemi* or gestational diabet* or stunted or wasted or stunting or wasting or failure to thrive or Vitamin D or rickets or Vitamin A or malnutrition or undernutrition or overweight or obes* or underweight or unhealthy diet* or diet* or body mass index or micronutrient* or arm circumference or breast fed* or breastfed* or breast feed* or breastfeed* or bottle fed* or bottle feed* or bottlefeed* or birth weight or fetal macrosomia or fetal weight or foetal weight or weaning or fetal growth or foetal growth or crown rump or infant food or infant formula or baby food or baby formula or Intrauterine growth restriction or Large for gestational age or Small for gestational age or foetal macrosomia or toddler food or toddler feed* or toddler eat* or toddler diet or preschool food or infant diet or preschool diet or preschool eat*).mp. [mp=title, abstract, heading word, drug trade name, original title, device manufacturer, drug manufacturer, device trade name, keyword, floating subheading word, candidate term word] |
| 67 | sexual crime/ |
| 68 | domestic violence/ or child neglect/ or child sexual abuse/ |
| 69 | exp human trafficking/ |
| 70 | exp attempted rape/ or exp statutory rape/ or exp acquaintance rape/ or exp rape/ or exp marital rape/ |
| 71 | partner violence/ |
| 72 | exp sexual abuse/ or exp physical abuse/ |
| 73 | battered woman/ |
| 74 | emotional abuse/ |
| 75 | gender based violence/ |
| 76 | courtship/ |
| 77 | extramarital sex/ or extramarital sexual intercourse/ |
| 78 | prostitution/ |
| 79 | exp unsafe sex/ |
| 80 | forced marriage/ |
| 81 | unsafe sex/ or barebacking/ or unprotected sex/ |
| 82 | (child marriage or forced marriage or early marriage or preferential feeding or sexual violence or domestic violence or intimate partner violence or sexual abuse or sexual exploitation or sexual assault or physical abuse or emotional abuse or emotional violence or psychological abuse or economic abuse or financial abuse or verbal abuse or violence against women or physical violence or psychological violence or sexual violence or economic violence or financial violence or dating violence or sexual coercion).mp. [mp=title, abstract, heading word, drug trade name, original title, device manufacturer, drug manufacturer, device trade name, keyword, floating subheading word, candidate term word] |
| 83 | epidemiology/ or causality/ or community sample/ or cross-sectional study/ or disease surveillance/ or health survey/ or incidence/ or prevalence/ |
| 84 | control group/ |
| 85 | crossover procedure/ |
| 86 | double blind procedure/ |
| 87 | intention to treat analysis/ or multivariate analysis/ or regression analysis/ |
| 88 | randomization/ or random digit dialing/ |
| 89 | observational study/ |
| 90 | single-case study/ or case study/ |
| 91 | case control study/ or clinical study/ or controlled study/ or hospital based case control study/ or population based case control study/ |
| 92 | cohort analysis/ |
| 93 | follow up/ |
| 94 | longitudinal study/ or "national longitudinal study of adolescent health"/ |
| 95 | prospective study/ |
| 96 | nutritional assessment/ |
| 97 | (Surveys and Questionnaires).mp. [mp=title, abstract, heading word, drug trade name, original title, device manufacturer, drug manufacturer, device trade name, keyword, floating subheading word, candidate term word] |
| 98 | (survey* or questionnaire* or cross sectional or nationally representative or longitudinal or mixed method*).mp. [mp=title, abstract, heading word, drug trade name, original title, device manufacturer, drug manufacturer, device trade name, keyword, floating subheading word, candidate term word] |
| 99 | 1 or 2 or 3 or 4 or 5 or 6 or 7 or 8 or 9 or 10 or 11 or 12 or 13 or 14 or 15 or 16 or 17 or 18 or 19 or 20 or 21 or 22 or 23 or 24 or 25 or 26 or 27 or 28 or 29 or 30 or 31 or 32 or 33 or 34 or 35 or 36 or 37 or 38 or 39 or 40 or 41 or 42 or 43 or 44 or 45 or 46 or 47 or 48 or 49 or 50 or 51 or 52 or 53 or 54 or 55 or 56 or 57 or 58 or 59 or 60 or 61 or 62 or 63 or 64 or 65 or 66 |
| 100 | 67 or 68 or 69 or 70 or 71 or 72 or 73 or 74 or 75 or 76 or 77 or 78 or 79 or 80 or 81 or 82 |
| 101 | 83 or 84 or 85 or 86 or 87 or 88 or 89 or 90 or 91 or 92 or 93 or 94 or 95 or 96 or 97 or 98 |
| 102 | 99 and 100 and 101 |

Database(s): **Global Health**

- Dates Searched:
  - 1910 to 2021 Week 30, n=720
  - 2021-2022 November 3, n=151

Search Strategy:

| **#** | **Searches** |
| --- | --- |
| 1 | malnutrition/ or nutritional disorders/ or cachexia/ or deficiency diseases/ or hunger/ or kwashiorkor/ or nutrition/ or protein energy malnutrition/ |
| 2 | rickets/ or vitamin d deficiency/ |
| 3 | starvation/ or deprivation/ or famine/ or fasting/ or underfeeding/ or undernutrition/ or underweight/ |
| 4 | overfeeding/ or feeding frequency/ or obesity/ or overeating/ |
| 5 | body composition/ or body fat/ or body lean mass/ |
| 6 | body weight/ or weight/ or body mass index/ or body measurements/ |
| 7 | chronic wasting disease/ |
| 8 | wasting disease/ or emaciation/ |
| 9 | infant nutrition/ or child nutrition/ or complementary feeding/ or infant feeding/ or infant foods/ |
| 10 | growth retardation/ |
| 11 | anthropometric dimensions/ |
| 12 | bottle feeding/ |
| 13 | breast feeding/ or exclusive breast feeding/ or human lactation/ or preweaning period/ or suckling/ or weaning/ |
| 14 | birth weight/ or low birth weight infants/ or postnatal development/ |
| 15 | amenorrhoea/ or maternal nutrition/ |
| 16 | nutritional state/ |
| 17 | nutrition policy/ or nutrition programmes/ |
| 18 | recommended dietary allowances/ or dietary guidelines/ or "u.s recommended daily allowances"/ or nutrient requirements/ |
| 19 | anaemia/ or nutritional anaemia/ or haemoglobin/ |
| 20 | iron deficiency/ |
| 21 | growth disorders/ |
| 22 | weight losses/ |
| 23 | weight gain/ |
| 24 | food intake/ or energy intake/ |
| 25 | failure to thrive/ |
| 26 | overweight/ |
| 27 | diet/ |
| 28 | nutrition/ |
| 29 | diabetes mellitus/ and pregnancy/ |
| 30 | thinness/ or weight reduction/ |
| 31 | anthropometric dimensions/ |
| 32 | height/ or height-weight ratio/ |
| 33 | menarche/ |
| 34 | dietary protein/ or protein intake/ |
| 35 | egg proteins/ or animal proteins/ or egg protein/ |
| 36 | meals/ or breakfast/ or lunch/ or snacks/ |
| 37 | food supply/ or food deserts/ or food security/ or food shortages/ |
| 38 | soft drinks/ or cola beverages/ or fruit juices/ or sugar sweetened beverages/ or "tea (beverage)"/ or vegetable juices/ |
| 39 | carbonation/ |
| 40 | food supplements/ |
| 41 | fast foods/ |
| 42 | iodine deficiency/ |
| 43 | vitamins/ or fat soluble vitamins/ or water-soluble vitamins/ or vitamin deficiencies/ |
| 44 | vitamin D.sh. |
| 45 | (caloric intake or energy restricted diets).sh. |
| 46 | portion size/ |
| 47 | appetite disorders/ or anorexia/ or bulimia/ or compulsive eating/ or overeating/ |
| 48 | epidemiological surveys/ or surveys/ or disease surveys/ |
| 49 | dietary surveys/ or nutrition surveys/ or nutritional assessment/ or diet studies/ |
| 50 | randomized controlled trials/ or clinical trials/ |
| 51 | cohort studies/ or longitudinal studies/ |
| 52 | longitudinal studies/ or retrospective studies/ or case studies/ |
| 53 | nutrition research/ |
| 54 | case-control studies/ or trials/ |
| 55 | sexual abuse/ or abuse/ or child abuse/ or sexual assault/ |
| 56 | domestic violence/ or spouse abuse/ |
| 57 | aggression/ |
| 58 | sexual discrimination/ |
| 59 | "rape (trauma)"/ or sexual assault/ |
| 60 | battered wom*.mp. [mp=abstract, title, original title, broad terms, heading words, identifiers, cabicodes] |
| 61 | (child marriage* or forced marriage* or early marriage*).mp. [mp=abstract, title, original title, broad terms, heading words, identifiers, cabicodes] |
| 62 | Preferential feeding.mp. [mp=abstract, title, original title, broad terms, heading words, identifiers, cabicodes] |
| 63 | sexual violence.mp. [mp=abstract, title, original title, broad terms, heading words, identifiers, cabicodes] |
| 64 | domestic violence.mp. [mp=abstract, title, original title, broad terms, heading words, identifiers, cabicodes] |
| 65 | intimate partner violence.mp. [mp=abstract, title, original title, broad terms, heading words, identifiers, cabicodes] |
| 66 | sexual abuse.mp. [mp=abstract, title, original title, broad terms, heading words, identifiers, cabicodes] |
| 67 | sexual exploitation.mp. [mp=abstract, title, original title, broad terms, heading words, identifiers, cabicodes] |
| 68 | (sexual assault or rape).mp. [mp=abstract, title, original title, broad terms, heading words, identifiers, cabicodes] |
| 69 | (physical abuse* or emotional abuse* or psychological abuse* or economic abuse* or financial abuse*).mp. [mp=abstract, title, original title, broad terms, heading words, identifiers, cabicodes] |
| 70 | verbal abuse*.mp. [mp=abstract, title, original title, broad terms, heading words, identifiers, cabicodes] |
| 71 | violence against women.mp. [mp=abstract, title, original title, broad terms, heading words, identifiers, cabicodes] |
| 72 | (physical violence or emotional violence or psychological violence or sexual violence or economic violence or financial violence).mp. [mp=abstract, title, original title, broad terms, heading words, identifiers, cabicodes] |
| 73 | dating violence.mp. [mp=abstract, title, original title, broad terms, heading words, identifiers, cabicodes] |
| 74 | sexual coercion.mp. [mp=abstract, title, original title, broad terms, heading words, identifiers, cabicodes] |
| 75 | gender based violence.mp. [mp=abstract, title, original title, broad terms, heading words, identifiers, cabicodes] |
| 76 | (iron deficien* or adrenarche or amenorrhea).mp. [mp=abstract, title, original title, broad terms, heading words, identifiers, cabicodes] |
| 77 | anemi*.mp. [mp=abstract, title, original title, broad terms, heading words, identifiers, cabicodes] |
| 78 | anaemi*.mp. [mp=abstract, title, original title, broad terms, heading words, identifiers, cabicodes] |
| 79 | gestational diabet*.mp. [mp=abstract, title, original title, broad terms, heading words, identifiers, cabicodes] |
| 80 | (stunted or wasted or stunting or wasting).mp. [mp=abstract, title, original title, broad terms, heading words, identifiers, cabicodes] |
| 81 | failure to thrive.mp. [mp=abstract, title, original title, broad terms, heading words, identifiers, cabicodes] |
| 82 | (Vitamin D or rickets).mp. [mp=abstract, title, original title, broad terms, heading words, identifiers, cabicodes] |
| 83 | malnutrition.mp. [mp=abstract, title, original title, broad terms, heading words, identifiers, cabicodes] |
| 84 | undernutrition.mp. [mp=abstract, title, original title, broad terms, heading words, identifiers, cabicodes] |
| 85 | (overweight or obes*).mp. [mp=abstract, title, original title, broad terms, heading words, identifiers, cabicodes] |
| 86 | underweight.mp. [mp=abstract, title, original title, broad terms, heading words, identifiers, cabicodes] |
| 87 | unhealthy diet*.mp. [mp=abstract, title, original title, broad terms, heading words, identifiers, cabicodes] |
| 88 | diet*.mp. [mp=abstract, title, original title, broad terms, heading words, identifiers, cabicodes] |
| 89 | body mass index.mp. [mp=abstract, title, original title, broad terms, heading words, identifiers, cabicodes] |
| 90 | micronutrient*.mp. [mp=abstract, title, original title, broad terms, heading words, identifiers, cabicodes] |
| 91 | arm circumference.mp. [mp=abstract, title, original title, broad terms, heading words, identifiers, cabicodes] |
| 92 | (breast fed or breastfed or breast feed* or breastfeed*).mp. [mp=abstract, title, original title, broad terms, heading words, identifiers, cabicodes] |
| 93 | bottle fed.mp. [mp=abstract, title, original title, broad terms, heading words, identifiers, cabicodes] |
| 94 | bottle feed*.mp. [mp=abstract, title, original title, broad terms, heading words, identifiers, cabicodes] |
| 95 | birth weight.mp. [mp=abstract, title, original title, broad terms, heading words, identifiers, cabicodes] |
| 96 | fetal macrosomia.mp. [mp=abstract, title, original title, broad terms, heading words, identifiers, cabicodes] |
| 97 | (fetal weight or infant weight).mp. [mp=abstract, title, original title, broad terms, heading words, identifiers, cabicodes] |
| 98 | foetal weight.mp. [mp=abstract, title, original title, broad terms, heading words, identifiers, cabicodes] |
| 99 | (weaning or fetal growth or foetal growth).mp. [mp=abstract, title, original title, broad terms, heading words, identifiers, cabicodes] |
| 100 | crown rump.mp. [mp=abstract, title, original title, broad terms, heading words, identifiers, cabicodes] |
| 101 | infant food.mp. [mp=abstract, title, original title, broad terms, heading words, identifiers, cabicodes] |
| 102 | (infant formula or baby food or baby formula).mp. [mp=abstract, title, original title, broad terms, heading words, identifiers, cabicodes] |
| 103 | Intrauterine growth restriction.mp. [mp=abstract, title, original title, broad terms, heading words, identifiers, cabicodes] |
| 104 | Large for gestational age.mp. [mp=abstract, title, original title, broad terms, heading words, identifiers, cabicodes] |
| 105 | Small for gestational age.mp. [mp=abstract, title, original title, broad terms, heading words, identifiers, cabicodes] |
| 106 | foetal macrosomia.mp. [mp=abstract, title, original title, broad terms, heading words, identifiers, cabicodes] |
| 107 | (maternal malnutrition or maternal underweight or maternal wasting).mp. [mp=abstract, title, original title, broad terms, heading words, identifiers, cabicodes] |
| 108 | (toddler food or toddler feed* or toddler eat* or toddler diet* or preschool food or infant diet or preschool diet or preschool eat*).mp. [mp=abstract, title, original title, broad terms, heading words, identifiers, cabicodes] |
| 109 | (survey* or questionnaire* or longitudinal or cross sectional or nationally representative or mixed method*).mp. [mp=abstract, title, original title, broad terms, heading words, identifiers, cabicodes] |
| 110 | 1 or 2 or 3 or 4 or 5 or 6 or 7 or 8 or 9 or 10 or 11 or 12 or 13 or 14 or 15 or 16 or 17 or 18 or 19 or 20 or 21 or 22 or 23 or 24 or 25 or 26 or 27 or 28 or 29 or 30 or 31 or 32 or 33 or 34 or 35 or 36 or 37 or 38 or 39 or 40 or 41 or 42 or 43 or 44 or 45 or 46 or 47 or 76 or 77 or 78 or 79 or 80 or 81 or 82 or 83 or 84 or 85 or 86 or 87 or 88 or 89 or 90 or 91 or 92 or 93 or 94 or 95 or 96 or 97 or 98 or 99 or 100 or 101 or 102 or 103 or 104 or 105 or 106 or 107 or 108 |
| 111 | 48 or 49 or 50 or 51 or 52 or 53 or 54 or 109 |
| 112 | 55 or 56 or 57 or 58 or 59 or 60 or 61 or 62 or 63 or 64 or 65 or 66 or 67 or 68 or 69 or 70 or 71 or 72 or 73 or 74 or 75 |
| 113 | 110 and 111 and 112 |

Database(s): **Ovid MEDLINE(R) and Epub Ahead of Print, In-Process, In-Data-Review & Other Non-Indexed Citations, Daily and Versions(R)**

- **Dates Searched:**
  - 1946 to July 23, 2021, n=1405
  - 2021-2022, November 3, n=178

Search Strategy:

| **#** | **Searches** |
| --- | --- |
| 1 | child nutrition disorders/ or malnutrition/ or deficiency diseases/ or rickets/ or protein deficiency/ or protein-energy malnutrition/ or severe acute malnutrition/ or kwashiorkor/ or starvation/ or overnutrition/ or obesity/ or obesity, morbid/ or pediatric obesity/ or wasting syndrome/ or infant nutrition disorders/ or bottle feeding/ or breast feeding/ or birth weight/ or fetal macrosomia/ or fetal weight/ or infant, low birth weight/ or infant, small for gestational age/ or infant, very low birth weight/ or infant, extremely low birth weight/ or weaning/ or prenatal nutritional physiological phenomena/ or fetal growth retardation/ or fetal nutrition disorders/ or crown-rump length/ or infant food/ or infant formula/ |
| 2 | nutrition policy/ or recommended dietary allowances/ |
| 3 | anemia, hypochromic/ or anemia, iron-deficiency/ |
| 4 | Growth Disorders/ |
| 5 | body weight changes/ or weight gain/ or gestational weight gain/ or weight loss/ or emaciation/ or cachexia/ or overweight/ or obesity/ or failure to thrive/ |
| 6 | Diet, Healthy/ |
| 7 | body mass index/ |
| 8 | body fat distribution/ or adiposity/ |
| 9 | Diabetes, Gestational/ |
| 10 | Thinness/ |
| 11 | anthropometry/ or cephalometry/ |
| 12 | growth/ or body height/ or body weight/ or weight gain/ or gestational weight gain/ or weight loss/ |
| 13 | adrenarche/ or menarche/ or Amenorrhea/ |
| 14 | dietary proteins/ or egg proteins, dietary/ or meat proteins/ or milk proteins/ |
| 15 | meals/ or breakfast/ or lunch/ or snacks/ |
| 16 | food supply/ or famine/ or food insecurity/ or food security/ |
| 17 | beverages/ or carbonated beverages/ or "fruit and vegetable juices"/ or sugar-sweetened beverages/ or tea/ |
| 18 | dietary supplements/ or fast foods/ or micronutrients/ or iodine/ or iron/ or vitamins/ or vitamin a/ or vitamin d/ or energy intake/ or caloric restriction/ or portion size/ or serving size/ or maternal nutritional physiological phenomena maternal nutritional physiological phenomena / or nutritional status/ |
| 19 | epidemiologic research design/ or control groups/ or cross-over studies/ or double-blind method/ or matched-pair analysis/ or random allocation/ or clinical studies as topic/ or clinical trials as topic/ or controlled clinical trials as topic/ or randomized controlled trials as topic/ or single-case studies as topic/ or observational studies as topic/ or epidemiologic studies/ or case-control studies/ or retrospective studies/ or cohort studies/ or follow-up studies/ or longitudinal studies/ or "national longitudinal study of adolescent health"/ or prospective studies/ or controlled before-after studies/ or cross-sectional studies/ or historically controlled study/ or interrupted time series analysis/ |
| 20 | "feeding and eating disorders"/ or anorexia nervosa/ or avoidant restrictive food intake disorder/ or binge-eating disorder/ or "feeding and eating disorders of childhood"/ or food addiction/ |
| 21 | sex offenses/ or child abuse, sexual/ or human trafficking/ or rape/ or domestic violence/ or spouse abuse/ or gender-based violence/ or intimate partner violence/ |
| 22 | Physical Abuse/ |
| 23 | Battered Women/ |
| 24 | Aggression/ |
| 25 | courtship/ or extramarital relations/ or sex work/ or unsafe sex/ |
| 26 | child marriage.mp. [mp=title, abstract, original title, name of substance word, subject heading word, floating sub-heading word, keyword heading word, organism supplementary concept word, protocol supplementary concept word, rare disease supplementary concept word, unique identifier, synonyms] |
| 27 | forced marriage.mp. [mp=title, abstract, original title, name of substance word, subject heading word, floating sub-heading word, keyword heading word, organism supplementary concept word, protocol supplementary concept word, rare disease supplementary concept word, unique identifier, synonyms] |
| 28 | early marriage.mp. [mp=title, abstract, original title, name of substance word, subject heading word, floating sub-heading word, keyword heading word, organism supplementary concept word, protocol supplementary concept word, rare disease supplementary concept word, unique identifier, synonyms] |
| 29 | Preferential feeding.mp. [mp=title, abstract, original title, name of substance word, subject heading word, floating sub-heading word, keyword heading word, organism supplementary concept word, protocol supplementary concept word, rare disease supplementary concept word, unique identifier, synonyms] |
| 30 | sexual violence.mp. [mp=title, abstract, original title, name of substance word, subject heading word, floating sub-heading word, keyword heading word, organism supplementary concept word, protocol supplementary concept word, rare disease supplementary concept word, unique identifier, synonyms] |
| 31 | domestic violence.mp. [mp=title, abstract, original title, name of substance word, subject heading word, floating sub-heading word, keyword heading word, organism supplementary concept word, protocol supplementary concept word, rare disease supplementary concept word, unique identifier, synonyms] |
| 32 | intimate partner violence.mp. [mp=title, abstract, original title, name of substance word, subject heading word, floating sub-heading word, keyword heading word, organism supplementary concept word, protocol supplementary concept word, rare disease supplementary concept word, unique identifier, synonyms] |
| 33 | sexual abuse.mp. [mp=title, abstract, original title, name of substance word, subject heading word, floating sub-heading word, keyword heading word, organism supplementary concept word, protocol supplementary concept word, rare disease supplementary concept word, unique identifier, synonyms] |
| 34 | sexual exploitation.mp. [mp=title, abstract, original title, name of substance word, subject heading word, floating sub-heading word, keyword heading word, organism supplementary concept word, protocol supplementary concept word, rare disease supplementary concept word, unique identifier, synonyms] |
| 35 | sexual assault.mp. [mp=title, abstract, original title, name of substance word, subject heading word, floating sub-heading word, keyword heading word, organism supplementary concept word, protocol supplementary concept word, rare disease supplementary concept word, unique identifier, synonyms] |
| 36 | physical abuse.mp. [mp=title, abstract, original title, name of substance word, subject heading word, floating sub-heading word, keyword heading word, organism supplementary concept word, protocol supplementary concept word, rare disease supplementary concept word, unique identifier, synonyms] |
| 37 | emotional abuse.mp. [mp=title, abstract, original title, name of substance word, subject heading word, floating sub-heading word, keyword heading word, organism supplementary concept word, protocol supplementary concept word, rare disease supplementary concept word, unique identifier, synonyms] |
| 38 | psychological abuse.mp. [mp=title, abstract, original title, name of substance word, subject heading word, floating sub-heading word, keyword heading word, organism supplementary concept word, protocol supplementary concept word, rare disease supplementary concept word, unique identifier, synonyms] |
| 39 | (Economic abuse or Financial abuse).mp. [mp=title, abstract, original title, name of substance word, subject heading word, floating sub-heading word, keyword heading word, organism supplementary concept word, protocol supplementary concept word, rare disease supplementary concept word, unique identifier, synonyms] |
| 40 | verbal abuse.mp. [mp=title, abstract, original title, name of substance word, subject heading word, floating sub-heading word, keyword heading word, organism supplementary concept word, protocol supplementary concept word, rare disease supplementary concept word, unique identifier, synonyms] |
| 41 | violence against women.mp. [mp=title, abstract, original title, name of substance word, subject heading word, floating sub-heading word, keyword heading word, organism supplementary concept word, protocol supplementary concept word, rare disease supplementary concept word, unique identifier, synonyms] |
| 42 | (physical violence or emotional violence or psychological violence or sexual violence or economic violence or financial violence).mp. [mp=title, abstract, original title, name of substance word, subject heading word, floating sub-heading word, keyword heading word, organism supplementary concept word, protocol supplementary concept word, rare disease supplementary concept word, unique identifier, synonyms] |
| 43 | dating violence.mp. [mp=title, abstract, original title, name of substance word, subject heading word, floating sub-heading word, keyword heading word, organism supplementary concept word, protocol supplementary concept word, rare disease supplementary concept word, unique identifier, synonyms] |
| 44 | sexual coercion.mp. [mp=title, abstract, original title, name of substance word, subject heading word, floating sub-heading word, keyword heading word, organism supplementary concept word, protocol supplementary concept word, rare disease supplementary concept word, unique identifier, synonyms] |
| 45 | iron deficien*.mp. [mp=title, abstract, original title, name of substance word, subject heading word, floating sub-heading word, keyword heading word, organism supplementary concept word, protocol supplementary concept word, rare disease supplementary concept word, unique identifier, synonyms] |
| 46 | anemi*.mp. [mp=title, abstract, original title, name of substance word, subject heading word, floating sub-heading word, keyword heading word, organism supplementary concept word, protocol supplementary concept word, rare disease supplementary concept word, unique identifier, synonyms] |
| 47 | anaemi*.mp. [mp=title, abstract, original title, name of substance word, subject heading word, floating sub-heading word, keyword heading word, organism supplementary concept word, protocol supplementary concept word, rare disease supplementary concept word, unique identifier, synonyms] |
| 48 | gestational diabet*.mp. [mp=title, abstract, original title, name of substance word, subject heading word, floating sub-heading word, keyword heading word, organism supplementary concept word, protocol supplementary concept word, rare disease supplementary concept word, unique identifier, synonyms] |
| 49 | (stunted or wasted or stunting or wasting).mp. [mp=title, abstract, original title, name of substance word, subject heading word, floating sub-heading word, keyword heading word, organism supplementary concept word, protocol supplementary concept word, rare disease supplementary concept word, unique identifier, synonyms] |
| 50 | failure to thrive.mp. [mp=title, abstract, original title, name of substance word, subject heading word, floating sub-heading word, keyword heading word, organism supplementary concept word, protocol supplementary concept word, rare disease supplementary concept word, unique identifier, synonyms] |
| 51 | (Vitamin D or rickets).mp. [mp=title, abstract, original title, name of substance word, subject heading word, floating sub-heading word, keyword heading word, organism supplementary concept word, protocol supplementary concept word, rare disease supplementary concept word, unique identifier, synonyms] |
| 52 | malnutrition.mp. [mp=title, abstract, original title, name of substance word, subject heading word, floating sub-heading word, keyword heading word, organism supplementary concept word, protocol supplementary concept word, rare disease supplementary concept word, unique identifier, synonyms] |
| 53 | undernutrition.mp. [mp=title, abstract, original title, name of substance word, subject heading word, floating sub-heading word, keyword heading word, organism supplementary concept word, protocol supplementary concept word, rare disease supplementary concept word, unique identifier, synonyms] |
| 54 | (overweight or obes*).mp. [mp=title, abstract, original title, name of substance word, subject heading word, floating sub-heading word, keyword heading word, organism supplementary concept word, protocol supplementary concept word, rare disease supplementary concept word, unique identifier, synonyms] |
| 55 | underweight.mp. [mp=title, abstract, original title, name of substance word, subject heading word, floating sub-heading word, keyword heading word, organism supplementary concept word, protocol supplementary concept word, rare disease supplementary concept word, unique identifier, synonyms] |
| 56 | unhealthy diet*.mp. [mp=title, abstract, original title, name of substance word, subject heading word, floating sub-heading word, keyword heading word, organism supplementary concept word, protocol supplementary concept word, rare disease supplementary concept word, unique identifier, synonyms] |
| 57 | diet*.mp. [mp=title, abstract, original title, name of substance word, subject heading word, floating sub-heading word, keyword heading word, organism supplementary concept word, protocol supplementary concept word, rare disease supplementary concept word, unique identifier, synonyms] |
| 58 | body mass index.mp. [mp=title, abstract, original title, name of substance word, subject heading word, floating sub-heading word, keyword heading word, organism supplementary concept word, protocol supplementary concept word, rare disease supplementary concept word, unique identifier, synonyms] |
| 59 | micronutrient*.mp. [mp=title, abstract, original title, name of substance word, subject heading word, floating sub-heading word, keyword heading word, organism supplementary concept word, protocol supplementary concept word, rare disease supplementary concept word, unique identifier, synonyms] |
| 60 | arm circumference.mp. [mp=title, abstract, original title, name of substance word, subject heading word, floating sub-heading word, keyword heading word, organism supplementary concept word, protocol supplementary concept word, rare disease supplementary concept word, unique identifier, synonyms] |
| 61 | nutrition assessment/ |
| 62 | nutrition surveys/ or diet surveys/ or "Surveys and Questionnaires"/ |
| 63 | breast fed*.mp. [mp=title, abstract, original title, name of substance word, subject heading word, floating sub-heading word, keyword heading word, organism supplementary concept word, protocol supplementary concept word, rare disease supplementary concept word, unique identifier, synonyms] |
| 64 | breastfed*.mp. [mp=title, abstract, original title, name of substance word, subject heading word, floating sub-heading word, keyword heading word, organism supplementary concept word, protocol supplementary concept word, rare disease supplementary concept word, unique identifier, synonyms] |
| 65 | bottle fed*.mp. [mp=title, abstract, original title, name of substance word, subject heading word, floating sub-heading word, keyword heading word, organism supplementary concept word, protocol supplementary concept word, rare disease supplementary concept word, unique identifier, synonyms] |
| 66 | birth weight.mp. [mp=title, abstract, original title, name of substance word, subject heading word, floating sub-heading word, keyword heading word, organism supplementary concept word, protocol supplementary concept word, rare disease supplementary concept word, unique identifier, synonyms] |
| 67 | fetal macrosomia.mp. [mp=title, abstract, original title, name of substance word, subject heading word, floating sub-heading word, keyword heading word, organism supplementary concept word, protocol supplementary concept word, rare disease supplementary concept word, unique identifier, synonyms] |
| 68 | fetal weight.mp. [mp=title, abstract, original title, name of substance word, subject heading word, floating sub-heading word, keyword heading word, organism supplementary concept word, protocol supplementary concept word, rare disease supplementary concept word, unique identifier, synonyms] |
| 69 | foetal weight.mp. [mp=title, abstract, original title, name of substance word, subject heading word, floating sub-heading word, keyword heading word, organism supplementary concept word, protocol supplementary concept word, rare disease supplementary concept word, unique identifier, synonyms] |
| 70 | weaning.mp. [mp=title, abstract, original title, name of substance word, subject heading word, floating sub-heading word, keyword heading word, organism supplementary concept word, protocol supplementary concept word, rare disease supplementary concept word, unique identifier, synonyms] |
| 71 | fetal growth.mp. [mp=title, abstract, original title, name of substance word, subject heading word, floating sub-heading word, keyword heading word, organism supplementary concept word, protocol supplementary concept word, rare disease supplementary concept word, unique identifier, synonyms] |
| 72 | foetal growth.mp. [mp=title, abstract, original title, name of substance word, subject heading word, floating sub-heading word, keyword heading word, organism supplementary concept word, protocol supplementary concept word, rare disease supplementary concept word, unique identifier, synonyms] |
| 73 | crown rump.mp. [mp=title, abstract, original title, name of substance word, subject heading word, floating sub-heading word, keyword heading word, organism supplementary concept word, protocol supplementary concept word, rare disease supplementary concept word, unique identifier, synonyms] |
| 74 | infant food.mp. [mp=title, abstract, original title, name of substance word, subject heading word, floating sub-heading word, keyword heading word, organism supplementary concept word, protocol supplementary concept word, rare disease supplementary concept word, unique identifier, synonyms] |
| 75 | infant formula.mp. [mp=title, abstract, original title, name of substance word, subject heading word, floating sub-heading word, keyword heading word, organism supplementary concept word, protocol supplementary concept word, rare disease supplementary concept word, unique identifier, synonyms] |
| 76 | baby food.mp. [mp=title, abstract, original title, name of substance word, subject heading word, floating sub-heading word, keyword heading word, organism supplementary concept word, protocol supplementary concept word, rare disease supplementary concept word, unique identifier, synonyms] |
| 77 | baby formula.mp. [mp=title, abstract, original title, name of substance word, subject heading word, floating sub-heading word, keyword heading word, organism supplementary concept word, protocol supplementary concept word, rare disease supplementary concept word, unique identifier, synonyms] |
| 78 | Intrauterine growth restriction.mp. [mp=title, abstract, original title, name of substance word, subject heading word, floating sub-heading word, keyword heading word, organism supplementary concept word, protocol supplementary concept word, rare disease supplementary concept word, unique identifier, synonyms] |
| 79 | Large for gestational age.mp. [mp=title, abstract, original title, name of substance word, subject heading word, floating sub-heading word, keyword heading word, organism supplementary concept word, protocol supplementary concept word, rare disease supplementary concept word, unique identifier, synonyms] |
| 80 | Small for gestational age.mp. [mp=title, abstract, original title, name of substance word, subject heading word, floating sub-heading word, keyword heading word, organism supplementary concept word, protocol supplementary concept word, rare disease supplementary concept word, unique identifier, synonyms] |
| 81 | breast feed*.mp. [mp=title, abstract, original title, name of substance word, subject heading word, floating sub-heading word, keyword heading word, organism supplementary concept word, protocol supplementary concept word, rare disease supplementary concept word, unique identifier, synonyms] |
| 82 | breastfeed*.mp. [mp=title, abstract, original title, name of substance word, subject heading word, floating sub-heading word, keyword heading word, organism supplementary concept word, protocol supplementary concept word, rare disease supplementary concept word, unique identifier, synonyms] |
| 83 | bottle feed*.mp. [mp=title, abstract, original title, name of substance word, subject heading word, floating sub-heading word, keyword heading word, organism supplementary concept word, protocol supplementary concept word, rare disease supplementary concept word, unique identifier, synonyms] |
| 84 | bottlefeed*.mp. [mp=title, abstract, original title, name of substance word, subject heading word, floating sub-heading word, keyword heading word, organism supplementary concept word, protocol supplementary concept word, rare disease supplementary concept word, unique identifier, synonyms] |
| 85 | foetal macrosomia.mp. [mp=title, abstract, original title, name of substance word, subject heading word, floating sub-heading word, keyword heading word, organism supplementary concept word, protocol supplementary concept word, rare disease supplementary concept word, unique identifier, synonyms] |
| 86 | toddler food.mp. [mp=title, abstract, original title, name of substance word, subject heading word, floating sub-heading word, keyword heading word, organism supplementary concept word, protocol supplementary concept word, rare disease supplementary concept word, unique identifier, synonyms] |
| 87 | toddler feed*.mp. [mp=title, abstract, original title, name of substance word, subject heading word, floating sub-heading word, keyword heading word, organism supplementary concept word, protocol supplementary concept word, rare disease supplementary concept word, unique identifier, synonyms] |
| 88 | toddler eat*.mp. [mp=title, abstract, original title, name of substance word, subject heading word, floating sub-heading word, keyword heading word, organism supplementary concept word, protocol supplementary concept word, rare disease supplementary concept word, unique identifier, synonyms] |
| 89 | toddler diet.mp. [mp=title, abstract, original title, name of substance word, subject heading word, floating sub-heading word, keyword heading word, organism supplementary concept word, protocol supplementary concept word, rare disease supplementary concept word, unique identifier, synonyms] |
| 90 | preschool food.mp. [mp=title, abstract, original title, name of substance word, subject heading word, floating sub-heading word, keyword heading word, organism supplementary concept word, protocol supplementary concept word, rare disease supplementary concept word, unique identifier, synonyms] |
| 91 | infant diet.mp. [mp=title, abstract, original title, name of substance word, subject heading word, floating sub-heading word, keyword heading word, organism supplementary concept word, protocol supplementary concept word, rare disease supplementary concept word, unique identifier, synonyms] |
| 92 | preschool diet.mp. [mp=title, abstract, original title, name of substance word, subject heading word, floating sub-heading word, keyword heading word, organism supplementary concept word, protocol supplementary concept word, rare disease supplementary concept word, unique identifier, synonyms] |
| 93 | preschool eat*.mp. [mp=title, abstract, original title, name of substance word, subject heading word, floating sub-heading word, keyword heading word, organism supplementary concept word, protocol supplementary concept word, rare disease supplementary concept word, unique identifier, synonyms] |
| 94 | 1 or 2 or 3 or 4 or 5 or 6 or 7 or 8 or 9 or 10 or 11 or 12 or 13 or 14 or 15 or 16 or 17 or 18 or 20 or 45 or 46 or 47 or 48 or 49 or 50 or 51 or 52 or 53 or 54 or 55 or 56 or 57 or 58 or 59 or 60 or 63 or 64 or 65 or 66 or 67 or 68 or 69 or 70 or 71 or 72 or 73 or 74 or 75 or 76 or 77 or 78 or 79 or 80 or 81 or 82 or 83 or 84 or 85 or 86 or 87 or 88 or 89 or 90 or 91 or 92 or 93 |
| 95 | 21 or 22 or 23 or 24 or 25 or 26 or 27 or 28 or 29 or 30 or 31 or 32 or 33 or 34 or 35 or 36 or 37 or 38 or 39 or 40 or 41 or 42 or 43 or 44 |
| 96 | 94 and 95 |
| 97 | 19 or 61 or 62 |
| 98 | (survey or questionnaire or cross sectional or nationally representative or longitudinal or mixed method*).mp. [mp=title, abstract, original title, name of substance word, subject heading word, floating sub-heading word, keyword heading word, organism supplementary concept word, protocol supplementary concept word, rare disease supplementary concept word, unique identifier, synonyms] |
| 99 | 97 or 98 |
| 100 | 94 and 95 and 99 |
