# Supplementary material for: Linkages between maternal experience of intimate partner violence and child nutrition outcomes: A rapid evidence assessment
Source: PLoS One. 2024 Mar 18;19(3):e0298364. doi: 10.1371/journal.pone.0298364 (PMC10947923; doi:10.1371/journal.pone.0298364)
Supplement: S1 Table — (DOCX) [file pone.0298364.s002.docx]

**S1 Table: Study Results**

| **Study** | **Adjusted Result(s)** | **Potential Confounders** | **Mediators** | **Moderators** | **Summary of findings/results** |
| --- | --- | --- | --- | --- | --- |
| **Fetal growth** | | | | | |
| Khan (2020) | AOR 1.23 (1.05 to 1.45)* p<0.05 | Adjusted for sociodemographic variables, Residence, Maternal education, Economic status, Employment status, Adverse maternal characteristics, Underweight mother, Overweight/obese mother, Taken ANC <4 times, Unwanted birth, Ever had a terminated pregnancy, Victim of intimate partner violence, Maternal high-risk fertility behaviours, Maternal age at birth <18 years, Maternal age at birth >34 years, Birth interval <24 months, Birth order >3, Maternal age at birth <18 years and birth interval <24 months, Maternal age at birth >34 years and birth interval <24 months, Birth order >3 and birth interval <24 months | No | No | Adverse maternal experiences increases the prevalence of LBW in infants. |
| Vo (2019) | Pre-term/low birthweight (n=160) Non-pre-term/low birthweight (n=939):  Domestic violence POR 1.44* (1.04–1.99)* p= 0.029*  Emotional violence POR 1.27 (0.85–1.91) p= 0.250  Physical violence POR 0.69 (0.37–1.28) p=0.237  Sexual violence POR 1.49* (1.01–2.19)* p= 0.046* | age groups, occupations, housing status, economic status, husband’s age group, and alcohol consuming habit. | No | No | Exposure to DV leads to higher risk of adverse birth outcomes |
| Eno (2014) | Comparing cases (IPV (in past year or during pregnancy) vs. controls on birth wright: Birth weight less than 2500: Relative risk 1.5 , chi2:16.05, p<.001; Birth weight  2500 − 2.999, Relative risk: 1.7, chi2: 35.85 p<0.001; Birth weight  3000 − 3499, Relative risk 0.8, chi2: 1.385, p=0.239. More than  3499, Relative risk 0.9, chi2: 0.111, p=0.739 | No | No | No | The adverse neonatal outcomes were consistent with previous reports of adverse neonatal outcome as a complication of domestic violence in pregnancy. The pathway of the direct effect of physical trauma from domestic violence encountered during pregnancy and leading to adverse obstetric outcome was not applicable in the present study, while the indirect pathway of late onset of prenatal care, late presentation in labor, and physical and emotional deprivation appear to be more relevant. |
| Nguyen Hoang (2016) | Emotional violence Model 1 AOR 1.5 (0.9–2.6)  Physical violence AOR 7.3 (3.2–17.1) Model 2 AOR 5.7 (2.2–14.9)  Sexual violence AOR 1.8 (0.9–3.7) | Model 1 adjusted for emotional violence, sexual violence  Model 2 adjusted for emotional violence, sexual violence, previous low birth weight, miscarriages, stillbirth, women's age, education, occupation, BMI, anemia status | No | No | IPV, specifically physical IPV, is common during pregnancy and is associated with increased risk of preterm birth and LBW |
| Assefa (2012) | Any physical IPV and LBW: 1.7 (1.12 2.48), p<.05, Sexual IPV only, not significant, 0.5 (0.22, 1.01) | Yes - in final model, included age, education, residence, wealth status, parity, MUAC, fertility desire, ANC, violence (included as variable as measuring all determinants) and time to walk to health facility | No | No | Experience of physical violence during pregnancy was found to predict LBW |
| Kana (2020) | Any form of IPV B −99 (−201 to 2)  Any form of emotional IPV B −94 (−202 to 15)  Any form of physical IPV B−162 (−267 to −58)  Any form of sexual IPV B −139 (−248 to −30)  Any two forms of IPV B −145 (−258 to −32)  All three forms of IPV B −223 (−368 to −77)  Exposure to any IPV 1–5 times B 50 (−80 to 179) p<0.01 >5 times B −172 (−279 to −64) Any form of emotional IPV 1–5 times B −11 (−143 to 121) p= 0.01 >5 times B −155 (−277 to −33) Any form of physical IPV 1–5 times B−112 (−219 to −4) p<0.01 >5 times B −380 (−553 to −206) Any form of sexual IPV 1–5 times B −48 (−174 to 77) p<0.01 >5 times B −262 (−401 to −123) | Maternal age, parity, education and household poverty level | No | No | Exposure to IPV is associated with reduced birth weight. Combining more than one form of IPV leads to lower BW. |
| Ferdos (2017) | Physical IPV AOR 3.01 (2.35–5.81)  Sexual IPV AOR 1.98 (1.23–4.15)  Both physical and sexual IPV AOR 4.05 (2.79–7.33) p=.1 | Maternal age, age at marriage, place of residence, maternal education, occupation,  pregnancy intention, participation in household decision-makings, number of antenatal checkup, maternal  BMI and maternal height. | No | No | Maternal lifetime IPV experience of IPV is positively associated with LBW children |
| Batool (2018) | Spousal violence and birth weights  Physical Violence OR 1.35 (0.96-1.89)* p<0.05 Emotional Violence OR 1.10 (0.84-1.44) Combined Forms of Violence OR 0.63 (0.40-1.00) Severe Violence OR 1.49 (1.04-2.13)* p<0.05 | Woman's education and wealth index | No | No | Experiencing IPV has a negative impact on weight. Severe violence has a particular negative impact |
| Berhanie (2019) | AOR, any IPV and adverse birth outcomes (LBW and preterm birth): 3.119 [1.470,6.618], p<.05; AOR, physical violence and adverse birth outcomes: 4.767 [2.515,9.034], p<.05; psychological violence and adverse birth outcomes (LBW and preterm birth): 1.423 [0.732,2.764], sexual violence and adverse birth outcomes (LBW and preterm birth): 0.798 [0.499,1.276]; controlling behaviours and adverse birth outcomes (LBW and preterm birth): 0.755[.479,1.191]. | Yes - adjustment for age of women, marital status, occupation, area of residency, monthly income, educational status of women, ANC follow up, number of ANC visits, gravidity, and planned intention. | No | No | The finding of this study also showed that women exposed to all type of intimate partner violence during pregnancy were three times more likely to experience LBW (AOR = 3.1; CI 95% [1.47,6.62]); It was observed that, women who had been exposed to physical IPV during pregnancy were five times more likely to experience LBW (AOR = 4.767; CI 95% [2.515,9.034]) |
| Pena (2002) | Outcome category No. of cases Adjusted OR 95% CI All LBW AOR 3.98 (1.70, 9.31) LBW, all preterm AOR 2.88 (0.92, 9.06) LBW, term, Growth Restricted AOR 2.82 (1.24, 6.42) LBW, all Acute Growth Restricted AOR 3.52 (1.68, 16.61) LBW, all Chronic Growth Restricted AOR 3.59 (1.22, 10.43) | Age, parity, smoking, and socioeconomic status | No | No | Physical IPV is associated with LBW. |
| Rahman (2021) | Logistic regression model of LBW  Model 1 OR (95% CI)  Any IPV 1.17 (1.01–1.36)** p<0.05 Any PV 1.15 (0.99–1.34)* p<0.1 Any EV 1.26 (1.04–1.54)** p<0.05 Any SV 1.01 (0.79–1.30)  Model 2 OR (95% CI) Any IPV 1.19 (1.02–1.37)** p<0.05 Any PV 1.16 (1.00 1.35)** p<0.05 Any EV 1.29 (1.06 1.56)*** p<0.01 Any SV 1.02 (0.79 1.31)  Linear model of birth weight Model 1 Beta Coeff. (95% CI) Any IPV -31.15 (-61.9 to -0.41)* p<0.1 Any PV -27.58 (-59.47 to -4.3) Any EV -48.74 (-91.35 to -6.13)** p<0.05 Any SV 0.48 (-56.80 to 57.76) Model 2 Beta Coeff. (95% CI) Any IPV -32.39 (-63.05 to -1.73)** p<0.05 Any PV -28.40 (-60.13 to 3.36)  Any EV -51.69 (-93.97 to -9.42)*** p<0.01 Any SV -6.26 (-62.77 to 50.26) | Model 1: Place of residence, wealth index, education, mother’s current age, and mother’s age at last birth Model 2: Further adjusts for any ANC visit, birth order, BMI, smoking status, food intake index, anemia, hypertension, and blood glucose | No | No | Experiencing lifetime IPV for mothers increases the risk of LBW in babies and reduced weight. Physical IPV is associated with LBW and reduced weight. Emotional IPV is associated LBW and reduced weight. |
| Abujilban (2017) | Comparing birth weights of children of women who experienced physical violence during pregnancy vs. not: significant difference between the two groups in babies’ weight, F(1,155) = 4.6, p = .03*, partial η2 = 0.03. | Yes - only controlled for women's age | No | No | There is a significant difference in newborn’s birth weight between the victims of violence and non-victims with a small effect size after controlling the effect of maternal age. Newborns of non-victims were significantly heavier than newborns of victims |
| Marimuthu (2019) | Nonphysical abuse by spouse Present AOR 3.6 (1.3-9.9) p<0.011 | Gender, Pregnancy Induced Hypertension, and perceived social support | No | Yes - explored association of violence and LBW by sex of child, stratified by parity. Found that the odds of female children having LBW was more among nulliparous mothers(OR = 2.9) when compared to multiparous mothers(OR = 1.2) | Experiencing nonphysical IPV for mothers increases the risk of LBW in babies |
| Devi Pun (2019) | AOR Model 1 (controlling for study site, age, education and parity): Any DV and low birthweight: 0.98 (0.60–1.34); Fear only and low birthweight: 1.37 (0.80–2.03); violence only and low birthweight: 1.25 (0.54–2.90); both fear and violence and low birthweight: 0.61 (0.24–1.64); Model 2: also controlling for geographical setting and family type: Any DV and low birthweight: 0.91 (0.61–1.36)l Fear only: 1.23 (0.77–2.01); Violence only: 1.3 (0.57–3.02): Both fear and violence: 0.62 (0.24–1.64) | Yes - Model 1 adjusted for study site, age, education and parity in the first model. Second model also adjusted for geographical setting and family type | No | Yes - stratified by parity | No significant associations between any type of violence and LBW |
| Abadi (2013) | Significant difference in physical abuse during pregnancy and birth weight; no significant association for verbal or sexual abuse | No | No | No | The women who reported physical abuse during pregnancy had lower infant birth weight |
| Valladares (2009) | Mean cortisol levels for 65 abused mothers was higher than for 82 non abused mothers (p<0.001), LBW was higher in abused mothers but details were not given except that p<001. all violence was grouped together. IPV during pregnancy was associated with increased PM cortisol level and BW was reduced by a mean of 79 g. (p<0.001)IPV during pregnancy was associated with increased PM cortisol levels and decreased gestational age at delivery and reduced BW (42g). IPV specifically w direct abdominal trauma (not defined) decreased gestational age at delivery and BW by 65gm. | Included 'low social resources' which was included in the model. (was a significant predictor of LBW) (documented using an instrument by Hanson which includes 2 components: social network, and social support. | Cortisol in the intermediary in the pathway from the exposure to the LBW outcome | No | IPV during pregnancy predicts increased PM salivary cortisol which is associated with decreased birth weight either through earlier delivery (lower gestational age at delivery) or through lower BW at term |
| Vachhani 2022 | Domestic Violence and Low birth weight baby n= 46, 19.91% (total n=231) Domestic Violence and Poor eating habit n=16 6.93% (total n=231) | None listed | No | No | Of the women affected by domestic violence 19% had children with LBW. The eating habits of women who have been exposed to domestic violence were poor for 6.93% of those children. |
| Tesfa 2021 | IPV and Fetal malnutrition 1.97 (1.52-2.56)*, p<0.05 | Variables not specifically listed but it is stated that all variables with a p value less than or equal to 0.25 were considered for multivariable logistic regression to avoid confounders | No | No | IPV, low placental weight, and small for gestational age are significant factors in fetal malnutrition as was found in this first study on fetal malnutrition in Ethiopia |
| Okunola 2021 | IPV and Birth weight aOR1.17 95% CI(0.49–2.8) p=0.72 | parity, income, body mass index, gestational age at delivery | No | No | IPV in pregnancy can have an impact on adverse birth outcomes in women in Nigeria. The association between IPV and LBW was not significant in this study. |
| Doke 2021 | Domestic Violence and Low Birth Weight APR 0.9(0.5-1.8) p=0.84 | parental consanguinity, pre-existing maternal illness at conception, heavy work in the last six months of pregnancy, tobacco consumption, alcohol consumption, exposure to a pesticide, domestic violence during pregnancy | No | No | Although domestic violence was not found to be a risk factor for LBW other risk factors were identified for adverse birth outcomes. Parental relationship, pre-existing maternal illness at conception, tobacco consumption and heavy work in the last six months of pregnancy, were all associated with one or more adverse outcomes. |
| Mezmur (2021) | (1) Adjusted Prevalence Ratio (APR) among teenage mothers for IPV during pregnancy amd LBW: 1.39 (0.99,1.96) (2) APR among adult mothers: 2.22 (1.26,3.90)* | No formal education, marital status, nuclear family, cheew khat, antennatal care attendence, iron folic acid supplement, eclampsia, APH, Mode of delivery, income level | No | Regression results were stratified by teenage participants and adult participants. | IPV during pregnancy was associated with LBW only among adult women. |
| Faramarzi (2005) | AOR physical violence during pregnancy and LBW: 3.1 (2.6, 4.2); AOR any violence during pregnancy and LBW: 3.7 (2.6, 5.1) | age, economic statue, obtainment of prenatal care for physical violence model; age, education, residence, economic statue and obtainment of prenatal care. for all violence model | No | No | LBW was associated with physical violence and any violence during pregnancy |
| Debele 2022 | IPV and LBW 3.77 (1.81, 7.88) * p<0.05 | age of the mothers, parity, gestational age at birth, pregnancy-related complication, additional meal intake, alcohol intake, ANC follow-up, hemoglobin level < 11, physically demanding work during the current pregnancy, food insecurity, MUAC less than 23 cm, and intimate partner violence | No | No | Low birth weight is associated with a number of factors including food insecurity and IPV among others. |
| Dolatian (2016) | Violence has indirect effect on LBW, through pregnancy-anxiety - any violence, during pregnancy | Not relevant - path analysis | Yes - path analysis tested violence--> pregnancy related anxiety --> low birth weight. | No | Direct and indirect relationships between LBW and socioeconomic status, social support, and psychological factors. |
| Lobato (2018) | Psychological IPV, past year, and IUGR: all models were significant Model I 1.13 (1.06–1.20) <0.001 Model II 1.13 (1.06–1.21) <0.001 Model III 1.13 (1.06–1.21) <0.001 Model IV 1.15 (1.07–1.23) <0.001 | In model I, the relationship between psychologic IPV and IUGR controlled for only physical IPV. Model II included significant variables representing socioeconomic, demographic, and reproductive characteristics, whereas model III further added significant variables representing stressful life events during pregnancy, life habits, and social support. Last, all dimensions (variables) were fitted in model IV, including significant variables related to prenatal care and medical complications during pregnancy. | No | No | Psychological IPV was found to be a significant risk factor for IUGR. Mediation effects by some covariates—in particular, physical IPV, maternal smoking, and prenatal care—could not be ruled out, and the total effect is potentially higher than the direct effect observed in the present study. |
| Kaye (2006) | ORs: DV during pregnancy vs. no domestic violence during pregnancy: Anemia: 1.33 (0.98–1.82); Preterm birth vs. term birth: 1.45 (1.08–1.96); Small for date birth vs. term birth: 1.21 (0.87–1.69); Birthweight category: Low vs. normal: 1.49 (1.16–1.92) . Adjusted relative risk: for low birthweight: 3.78 (2.86–5.00); Population attributable risk: 0.41 (0.19–0.57). OR for low birthweight, adjusting for age, parity, number of living children, pregnancy timing and domicile: 1.66 (1.07–2.56) | Relative and attributable risk models adjusted for Adjusted for age, parity, number of living children, nature of prior pregnancy, pregnancy planning, domicile and household decision making. Multivariate logistic regression model included age, parity, number of living children, pregnancy timing and domicile | No | Yes- stratified for age when comparing birthweight between women exposed to DV and not exposed, as maternal age might confound the relationship between domestic violence and risk of LBW. | A significant proportion of LBW and antepartum hospitalization in this cohort was attributable to IPV, an effect possibly because of increased number of pregnancy-related complications among women reporting domestic violence. After adjusting for age and other variables, domestic violence was significantly associated with risk of LBW delivery. |
| Sigalla (2017) | IPV and preterm birth: AOR: Physical violence, Adjusting for previous history of miscarriage and preterm birth: AOR = 2.7; 95% CI 1.2–5.9); adjusting for women’s age, education level, occupation, and alcohol consumption, AOR = 2.8; 95% CI 1.3–6.5); Physical violence, adjusting for all confounders: 2.9 (1.3–6.5); IPV and LBW: Adjusting for previous history of miscarriage, preterm birth and LBW: AOR = 3.1; 95% CI 1.3–7.0; adjusting for women’s age, education level, occupation, and alcohol consumption, AOR = 3.4; 95% CI 1.4–7.9; Physical violence, adjusting for all confounders: AOR = 3.2; 95% CI: 1.3–7.7 | Adjusted for previous history of miscarriage and preterm birth, women's age, education level, occupation and alcohol consumption | No | Yes - stratified on previous adverse pregnancy outcome, found that among women with previous adverse pregnancy outcome, AOR is much higher: 4.5 (1.5–13.7) for preterm birth and 4.8 (1.6–14.8) for LBW | The results indicate that women who are exposed to violence during pregnancy are at significantly increased risk for giving birth preterm and/or having a low birth weight child. Women with previous adverse pregnancy outcome have an even higher risk of both IPV and recurrent PTB and LBW. |
| Musa (2021) | Low birth weight: Any IPV: 1.37(1.73, 2.57); Physical IPV: 2.25(1.23, 4.11); Emotional IPV: 2.15(1.20, 3.84); Preterm birth: Any IPV: 1.62(1.02, 2.58), Physical IPV: 1.20(0.72, 1.99); Emotional IPV: 1.45(0.88, 2.39) p<0.05 | Yes - ethnicity, age, occupation, education, average family income, pre-eclampsia, antepartum hemorrhage, history of stillbirth, parity, khat chewing | No | No | The findings of this study indicated that women who experienced any IPV during their most recent pregnancy had an increased likelihood of delivering prematurely and having a low birth weight infant. |
| Chandra 2021 | Violence During Pregnancy and Low birth weight in females OR 95%CI 2.48 (0.810, 7.581) p=0.112 Violence During Pregnancy and Low birth weight in Males OR 95%CI 0.45 (0.108, 1.898) p=0.279 | controlled for BMI, parity  and antenatal health care utilization | No | No | There was no statistically significant relationship between IPV during pregnancy and LBW in this study for males or females, although it was closer to a suggestion in female children |
| Shamu (2018) | Past 12 month IPV (any type) and history of negative pregnancy outcome (LBW, stillbirth, preterm birth) in most recent pregnancy - none significantly associated. AORs, adjusted for age, education, marital status, HIV status and history of smoking, violence or alcohol use. IPV during pregnancy and recent low birth weight: Emotional IPV: 1.79 (1.26–2.52) p=0.001, IPV during pregnancy and any recent negative outcome (LBW, premature baby. c section), 1.38 1.03–1.83 p=0.026. | Yes - age, education, marital status, HIV status and history of smoking, violence or alcohol use. | No | No | Experiencing emotional (but not physical or sexual) IPV during pregnancy was associated with a LBW baby. Emotional IPV was also associated with any negative outcome from the recent pregnancy. Only emotional violence was associated with low birth weight However, since emotional violence does not usually occur in isolation, physical and sexual abuse may also have contributed towards low birth weight. |
| Laelago (2017) | Exposure to any IPV during pregnancy and LBW: AOR=14.3 (5.1,40.7) | Yes - age of women, number of pregnancy, planned pregnancy, educational status of women and monthly income, | No | No | Significant association between intimate partner violence during pregnancy and low birth weight of newborn. |
| Shamu (2018) | Past 12 month IPV (any type) and history of negative pregnancy outcome (LBW, stillbirth, preterm birth) in most recent pregnancy - none significantly associated. AORs, adjusted for age, education, marital status, HIV status and history of smoking, violence or alcohol use. IPV during pregnancy and recent low birth weight: Emotional IPV: 1.79 (1.26–2.52) p=0.001, IPV during pregnancy and any recent negative outcome (LBW, premature baby. c section), 1.38 1.03–1.83 p=0.026. | Yes - age, education, marital status, HIV status and history of smoking, violence or alcohol use. | No | No | Experiencing emotional (but not physical or sexual) IPV during pregnancy was associated with a LBW baby. Emotional IPV was also associated with any negative outcome from the recent pregnancy. Only emotional violence was associated with low birth weight However, since emotional violence does not usually occur in isolation, physical and sexual abuse may also have contributed towards low birth weight. |
| Jaraba (2019) | AOR of violence during pregnancy and LBW: 1.03 (0.775–1.376). | Yes - education, age, place of residence, partner’s education level, geographic region of residency, age of first intercourse, prenatal care, numbers of prenatal care appointments, cigarette smoking, alcohol consumption, unwanted pregnancy, and iron and folic acid supplementation. | No | Yes - stratified for age (below 35 and above) | Contrary to the international literature, this study does not reveal any relation between violence during pregnancy and low birth weight. Some studies, especially those conducted in Latin America and in low-income countries, have reported that women who have suffered violence during pregnancy have up to a four times higher risk of having newborns with low birth weight compared with women who have not suffered violence |
| Alemu (2019) | IPV during pregnancy and LBW: 2.1(1.1-3.9), p=.025 | Yes - occupation, residence, family size, pregnancy was planned, ANC visit, parity, birth interval | No | No | Mother who had suffered any IPV during pregnancy were two times more likely to have low birth weight babies as compared to mothers who didn’t have any violence |
| Mahmoodi (2019) | Any violence during pregnancy and low birth weight: 1.89 (1.06-3.36), significant | No - not relevant, path analysis | Yes - path analysis; explored relationship of violence through depression | No | There was also a significant correlation between domestic violence and the low birth weight in this study. |
| Nojomi (2006) | Significant association between physical violence during pregnancy and low birth weight | Yes - not specified | No | No | After controlling for confounders, there was a significant association between physical violence and premature rupture of membranes (P = 0.005), low birth weight (P = 0.005) and preterm birth (P =0.003). |
| Mezzavilla (2016) | Global physical violence, past 12 months, and LBW: AOR 3.69, 1.57-8.66; severe assault vs. not physical violence AOR: 4.83, 1.82-12.8 | Yes - Controlled for environmental conditions, number of friends and relatives, maternal age, gestational weight gain and hypertension, and parity | No | No | Women who experience IPV are almost four times more likely to have children with low birth weight than women who do not. Additionally, as PIPV severity increases, the odds of having a LBW child also increase, regardless of environmental conditions, maternal age, gestational weight gain, social network and support, parity, and hypertension. |
| Nejatizade (2017) | statistically significant relationship between physical violence and low birth weight (LBW), which is of fetal outcomes (p=0.033). The results showed that there was a significant relationship between physical violence and LBW (p<0.000, r=0.164), but there was no statistically significant relationship between psychological and sexual violence with other variables related to fetal outcome. (recall period not specified) | Yes - not clearly specified | No | No | Statistically significant relationship between physical violence and low birth weight (LBW) (p=0.033) and between physical IPV and LBW, but there was no statistically significant relationship between psychological and sexual IPV with other variables related to fetal outcome. |
| Nunes (2011) | Violence during pregnancy and LBW: Model 1: Only psychological: 0.95 (0.41–2.19); Only physical: 2.48 (0.90–6.86); both: 3.02 (1.41–6.46). Model 2: only psychological: 0.57 (0.23–1.45); Only physical: 2.71 (0.75–9.77); Both: 2.39 (1.29–4.39); Model 3: Only psychological: 0.44 (0.16–1.16), Only physical: 3.19 (1.26–8.08); Both: 2.18 (1.16–4.08) | Including only family income and adequate number of prenatal visits (Model 1) statistically significant association for birth weight with categories of violence was seen only for physical plus psychological violence; including additionally length of pregnancy (Model 2), the association decreased in magnitude, but remained statistically significant; when adjusting additionally for gestational weight gain (Model 3), further decrease was seen, but remained statistically significant. | No | No | Women suffering psychological and physical IPV during pregnancy had a threefold increased risk of delivering a baby weighing <2500 g compared to those not suffering any type of violence. Adjusting simultaneously for family income, adequate number of prenatal visits, length of pregnancy and gestational weight gain, women suffering psychological and physical IPV during pregnancy still presented increased risk of delivering a LBW baby (RR 2.18; 95% CI 1.16–4.08%). |
| Nasreen (2019) | Risk ratio ever physical abuse and LBW: 8.39 (4.73–14.89), risk ratio physical abuse during pregnancy and LBW: 2.96 (1.20–7.33) | No, not for the risk ratios | No | Yes - tested if west or east coast residence moderated relationships | Physical IPV was emerged a risk factor for LBW and PTB in the west coast. |
| Moraes (2011) | Severe physical IPV during pregnancy and EBF: Model 1: 1.30 (1.01, 1.65), Model 2: 1.17 (0.89, 1.53) | Yes- The demographic and reproductive dimensions comprised ‘age’ (mothers and children), ‘sex’ (child), ‘number of children in the household’ (per mother) and the ‘mother’s desire to get pregnant’. The lifestyle dimension was represented by the current tobacco smoking habits (yes/no) and alcohol and illicit drug use during gestation. Alcohol (mis)use, illicit drug use. The consumption of at least one illicit substance (marijuana, cocaine or solvents) defined a positive case | Yes - tested child health, utilization of health services and maternal mental health. The first dimension was represented by the ‘mother’s perception regarding the child’s health status’ and ‘gestational age’ at birth. The use of health services was characterized by the ‘gestational age when the mother started her prenatal followup’, ‘number of prenatal health-care visits’ and ‘quality of maternity care’, the latter typified by whether the hospital in which the child was born adhered to a state-sponsored pro-breast-feeding programme (Baby-Friendly Hospital Initiative (BFHI))(24). Depicting the third mediating dimension, ‘maternal self-esteem’ was assessed through a Portuguese version of the Rosenberg scale as potential mediators | No | The association between SPVP and early cessation of EBF remained statistically significant even after allowing for confounders (Model I). Yet, when the variables representing ‘child health’, ‘utilization of health services’ and ‘degree of maternal self-esteem’ were introduced as possibly intervening in the process, the hazard ratio reduced and lost significance. |
| Ribeiro (2021) | IPV during pregnancy and EBF outcomes: No significant association between combined violence and EBF to 6 months; significant association between combined violence and continued breastfeeding to 1 year; was higher in cases of recurrent violence (assessed by violence before/during pregnancy, recurrent psychological/physical/sexual violence during pregnancy, recurrent psychological violence during pregnancy, and recurrent physical/sexual violence during pregnancy). | Yes, but exact ones included in models not indicated | No | No | No differences in exclusive breastfeeding duration in mothers exposed or not to any kind of IPV. However, the risk of breastfeeding cessation before 12 months of life was higher in cases of recurrent violence (assessed by violence before/during pregnancy, recurrent psychological/ physical/sexual violence during pregnancy, recurrent psychological violence during pregnancy, and recurrent physical/sexual violence during pregnancy). When compared to those mothers who did not report violence, the risk of not breastfeeding their infants until they were 12 months old was 39% higher when violence occurred before pregnancy and persisted during pregnancy; 46% higher for recurrent violence during pregnancy; 44% higher for recurrent psychological violence only during pregnancy; and 55% higher for recurrent physical/sexual violence during pregnancy |
| Caprara (2020) | Cox regression analysis, any violence and early complementary feeding, no association, 1.21 [0.66; 2.23] 0.535 | Yes - maternal education, marital status | No | No | In the present study, no significant association was found between early complementary feeding and domestic violence during pregnancy. Thus, there was no relationship between domestic violence against pregnant women and early complementary feeding, since violence was only a risk factor for the early complementary feeding in the univariate analysis, but was not significant when adjusted for other factors |
| **Infant feeding** | | | | | |
| Frith (2017) | Any lifetime DV (n 1593) UHM (n 824) aHR 1·13* (1·01, 1·26) BFC (n 769) aHR 0·99 (0·88, 1·11) p= 0·05  Any physical DV (n 597) UHM (n 313) aHR 0·98 (0·86, 1·12) BFC (n 284) aHR1·06 (0·92, 1·23) p= 0·68 Any sexual DV (n 639) UHM (n 332) aHR 1·15* (1·01, 1·31) BFC (n 307) aHR 1·01 (0·88, 1·16) p= 0·08  Any emotional DV (n 766) UHM (n 400) aHR1·02 (0·91, 1·15) BFC (n 366) aHR1·12 (0·99, 1·27) p= 0·41 Any controlling behaviour (n 1038) UHM (n 528) aHR1·10 (0·99, 1·23) BFC (n 510) 0·95 (0·84, 1·06) p= 0·05 | Maternal education, Socio-economic status and BMI | Exclusive breastfeeding counseling | No | EBF counseling is important to extend duration up to 6mo as is recommended by the WHO. Those participants who had lifetime exposure to DV and were in the EBF counseling group breastfeed longer than those who had the general health counseling. |
| Tran (2020) | Early initiation of breastfeeding:  Controlling behavior OR 0.87 (0.7,1.07)  Emotional violence OR 0.87 (0.72,1.05)  Physical violence OR 0.94 (0.76,1.16)  Sexual violence OR 0.95 (0.75,1.2)  All violence OR 0.91 (0.65,1.28)  Exclusive breastfeeding:  Controlling behavior OR 0.72 (0.56, 0.92)  Emotional violence OR 0.66 (0.53, 0.82)  Physical violence OR 1.04 (0.82,1.33)  Sexual violence OR 1.01 (0.77,1.32)  All violence OR 0.71 (0.54,0.93)  Optimal breastfeeding practice:  (b = -0.05, P < 0.05) and low maternal autonomy, and breastfeeding practices (b= -0.08, P < 0.05) | Maternal common disorder, age, education, parity, BMI, C-section, household SES, food security, child age, gender and geographical clustering | Maternal common mental disorder was included in an alternate pathway | No | IPV has an adverse effect on breastfeeding practices and combined interventions can have an impact on IPV and nutrition |
| Kjerulff Madsen (2019) | IPV in Current relationship Premature termination of exclusive breastfeeding (n = 577)  At least one type of IPV n= 326 AOR 1.61 (1.26 ; 2.05)  Emotional IPV n=297 AOR 1.61 (1.26 ; 2.07)  Physical IPV n=67 AOR 1.53 (1.01 ; 2.31)  Sexual IPV n=111 AOR 1.50 (1.07 ; 2.09)  All three types of IPV n=39 AOR 1.93 (1.11 ; 3.34)  IPV during pregnancy At least one type of IPV n=182 AOR 1.25 (0.96 ; 1.62)  Emotional IPV n=131 AOR 1.23 (0.91 ; 1.65)  Physical IPV n=42 AOR 1.68 (1.00 ; 2.82)  Sexual IPV n=97 AOR 1.35 (0.96 ; 1.91)  All three types of IPV n=23 AOR 2.87 (1.27 ; 6.46) | maternal age, education, HIV status, alcohol use during pregnancy and parity | No | Yes - stratified by child sex, maternal depression status, maternal age, maternal HIV status, maternal alcohol intake during pregnancy, maternal education | Increased and multiple types of IPV increase the odds of cessation of Exclusive breastfeeding before 6mo |
| Hasselmann (2016) | Relative risk of early cessation of breastfeeding (before 3 months), any IPV, past 12 months: 1.56 0.96-2.52 (not significant); severe IPV: 2.10 1.27-3.48 | Yes - sex, child’s age (in days), birth weight, maternal schooling, maternal age, and household assets 1 | No | No | The results of this study show that children of women who experienced physical violence were at greater risk of early interruption of exclusive breastfeeding, both in the second month of life, and in the following month, even after controlling for potential confounders. |
| Caleyachetty (2019) | Early initiation of breastfeeding  Physical violence AOR 0.90 (0.88,0.93)  Sexual violence AOR 0.84 (0.80,0.88)  Emotional violence AOR 0.90 (0.87,0.93)  All violence AOR 0.88 (0.86,0.91)  p<0.001 Exclusive breastfeeding  Physical violence AOR 0.86 (0.81,0.92) p<0.001 Sexual violence AOR 0.88 (0.79,0.97) p<0.001 Emotional violence AOR 0.88 (0.82, 0.95) p=0.009  All violence OR 0.87 (0.82,0.92) p=0.001 | mother’s age, mother’s level of education, household wealth, rural or urban residence, child’s age, and child’s sex adjusted to 3 types of IPV | No | Yes - stratified analysis by region and child's sex | Exposure to IPV in mothers impacts breastfeeding recommendation adoption |
| Ariyo (2021) | IPV and EBF complete cases p<0.05 Psychological IPV Model 1 AOR 0.73 (0.53, 1.01)  Physical IPV Model 2 AOR 0.69** (0.49, 0.98)  Sexual IPV Model 3 AOR 0.94 (0.60, 1.46)  Any form of IPV Model 4 AOR 0.82 (0.61, 1.11)  IPV frequency score Model 5 AOR 0.95** (0.90, 1.00) IPV and EBF Multiple imputation p<0.05 Psychological IPV Model 1 AOR 0.66** (0.47, 0.92) Physical IPV Model 2 AOR 0.63** (0.44, 0.90)  Sexual IPV Model 3 AOR 0.94 (0.62, 1.41) Any IPV Model 4 AOR 0.74** (0.55, 1.00) IPV frequency score Model 5 AOR 0.95** (0.90, 1.00) | Age, Education, Age (Child), Employed,  Parity, Antenatal, Perceived Birthweight, Education (Husband), Rural, Family wealth Index, number of children in household under 5 | No | No | Maternal exposure to IPV during pregnancy and postpartum has a negative association with the child being exclusively breastfed |
| Tiwari (2018) | Spousal ever emotional Violence and Breastfeeding (<1 month)  Plus confounders 1.19 (0.93,1.53)  Plus other violence 1.20 (0.92,1.57) | Age of the women, level of education of women and of their partners, employment status of the women, religion, caste, wealth quintile that the selected women’s household belonged to, and the presence of other forms of spousal violence, either physical or sexual | No | No | Emotional partner abuse or control is not associated with short duration of breastfeeding. Emotional abuse and control are both associated with other birth outcomes that do not relate to nutrition outcomes such as number of children born, termination of pregnancy and first birth |
| Boyce (2017) | Relationship between IPV and Breastfeeding  Early initiation of breastfeeding  Physical only AOR 0.81 (0.71–0.93)** p<0.01 Sexual only AOR 0.52 (0.36–0.76)*** p<0.001 Physical and sexual AOR 0.83 (0.67–1.01) Exclusive breastfeeding Physical only AOR 0.83 (0.71–0.96)* p<0.05 Sexual only AOR 0.74 (0.49–1.12) Physical and sexual AOR 0.92 (0.75–1.15) | residence in an Ananya program district, age of mother, age of mother at marriage, household wealth index, mother’s education, husband’s education, religion/caste status, gender of focal child, parity of mother, prior neonatal death or stillbirth, antenatal care visits, skilled birth attendant (SBA) at birth of focal child, age of focal child, and visits of community health worker in late pregnancy | No | No | IPV is largely associated with poor postnatal health practices in particular breastfeeding. It was negatively associated with healthy breastfeeding. |
| Young (2020) | Domestic violence associated early initiation of breastfeeding Domestic violence (last 12 months) AOR 1.14 (0.91, 1.42) p=0.2510 Domestic violence associated early initiation of prelacteal feeding Domestic violence (last 12 months) AOR 1.08 (0.88, 1.34) p = 0.4610 Domestic violence associated early initiation of exclusive breastfeeding Domestic violence (last 12 months) AOR 0.73** (0.60,0.90) p=0.0020 | religion, caste, child age, child sex, maternal age, socio-economic status, and food insecurity | No | No | IPV was associated with lower exclusive breastfeeding. |
| Zureick-Brown (2015) | Ever any IPV = 1  Breastfed immediately after birth (within 1 h) 1.04 (0.82, 1.33) p=0.717 Drank from a bottle with a nipple in the past 24 h 1.18 (0.83, 1.67) p=0.367 Fed liquids in the prior 24 h 1.32 (1.04, 1.66)* p=0.021 Fed solids in the prior 24 h 1.31 (0.89, 1.94) p=0.176 Exclusively breastfed in the prior 24 h 0.78 (0.62, 0.98)* p=0.034 Ever any physical or sexual IPV = 1  Breastfed immediately after birth (within 1 h) 1.04 (0.82, 1.32) p=0.756 Drank from a bottle with a nipple in the past 24 h 1.33 (0.93, 1.91) p=0.121 Fed liquids in the prior 24 h 1.37 (1.08, 1.75)* p=0.011 Fed solids in the prior 24 h 1.50 (1.01, 2.23)* p=0.046 Exclusively breastfed in the prior 24 h 0.74 (0.58, 0.95)* p=0.017 | Adjusted for stratified, cluster sample design, urban/rural residence, household wealth quintile, maternal age in years, child’s age in months, parity exclusive of index child, mother’s  completed grades of schooling, the difference in completed grades between the mother and her husband, mother’s relationship to household  head, gender of child, maternal religion, and region of residence | No | No | Mothers exposed to IPV may not be able to exclusively breastfeed due to psychological or physical factors as a result of abuse and need complementary foods. Mothers exposed to IPV may also not know the guidelines for exclusive breastfeeding. |
| Woldetensay 2021 | IPV and infant feeding practices beta= −0.208, CI( −0.337, −0.080)* p=0.001 SE=0.065 | Not stated | No | No | In Ethiopia the study shows poor infant feeding practices. In particular there was a relationship between depressive symptoms and infant feeding practices. IPV was negatively associated with infant feeding practices, along with postnatal depressive symptoms. Infant feeding practices were positively associated with social factors, these being social support perceived by the mother and social participation. |
| Tsedal (2021) | AOR: children of mothers who had intimate partner violence had 65% lesser odds of getting a minimum acceptable diet as compared to those who were children of women who did not have an intimate partner violence (AOR: 0.35; 95% CI: 0.16, 0.77) p,0.052.85 fold likelihood of not meeting a minimally acceptable diet among children whose mothers were exposed to IPV during the prior 12 months after adjusting for multiple factors. | Yes - residence (urban/ rural), maternal education, wealth index, presence of under 5 children in house, currently breastfeeding, fertilty desire, work status, child delivered in health facility, media exposure | No | No | Maternal exposure to IPV was associated with a lower proportion of children receiving a minimum acceptable diet (2.85 fold likelihood of not meeting a minimally acceptable diet among children whose mothers were exposed to IPV during the prior 12 months after adjusting for multiple factors). |
| Walters (2021) | Note: Adjusted results were only presented if bivariate were significant. IPV and delayed initiation of breastfeeding: Malawi: Emotional ever: 1.37 (1.05,1.80), p<.05, Sexual ever: 1.55 (1.14,2.10), p<.01; Emotional - never vs. yes but not in past 12 months: 1.56 (0.90,2.68), Emotional never vs. often or sometimes, 1.34 (0.99,1.79); Sexual: Never vs. Yes but not in past 12 months: 1.83 (0.98,3.42), Sexual never vs. Often or sometimes, 1.49 (1.08,2.06), p<.05. Tanzania: Physical ever: 1.44 (1.24,1.69), p<.0001; Emotional ever: 1.43 (1.22,1.68), p<.0001; Sexual ever: 1.30 (1.04,1.62), p<.05; Physical Never vs. yes but not in past 12 months: 1.38 (1.16,1.65), p<.001; Physical Never vs. often or sometimes: 1.55 (1.23,1.97), p<.001; Emotional: Never vs. yes but not in past 12 months: 1.43 (1.20,1.69), p<.001; Emotional never vs. often or sometimes: 1.45 (1.08,1.95), p<.05; Zambia: Physical ever: 1.13 (0.98,1.30); Emotional ever: 1.16 (0.99,1.02); Sexual ever: 1.28 (1.06,1.54), p<.05; Controlling behaviours ever: 1.28 (1.07,1.53), p<.01; Physical never vs. yes but not in past 12 months: 1.11 (0.96,1.37); Physical never vs. sometimes or often: 1.14 (0.91,1.34); Emotional never vs. yes but not in past 12 months: 1.35 (0.98,1.33); Emotional never vs. often or sometimes: 1.10 (0.92,1.84); Sexual never vs. yes but not in past 12 months: 1.55 (1.06,2.27), p<.05; Sexual never vs. sometimes or often: 1.22 (1.01,1.49), p<.05; IPV and non-exclusive breastfeeding: Malawi: Sexual ever: 1.90 (1.05,3.45), p<.05; Physical never vs. yes but not in past 12 months: 1.92 (0.99,3.72); Physical never vs. often or sometimes: 1.04 (0.46,2.32); Sexual never vs. yes but not in past 12 months: 1.34 (0.37,4.81); Sexual never vs. sometimes or often: 2.07 (1.08,3.96), p<.05; Tanzania: Controlling behaviours: 1.38 (0.96,1.99); Physical never vs. yes but not in past 12 months: 0.60 (0.35,1.02), Physical never vs. sometimes or often: 1.43 (0.98,2.09); Emotional never vs. yes but not in past 12 months: 0.38 (0.18,0.78), p<.01; Emotional never vs. often or sometimes: 1.07 (0.73,1.55); Zambia: Physical ever: 1.82 (1.31,2.51), p<.001; Sexual ever: 1.72 (1.18,2.50), p<.01; Emotional ever: 1.75 (1.15,2.67), p<.01; Physical never vs. yes but not in past 12 months: 1.52 (0.99,2.33), Physical never vs. often or sometimes, 2.11 (1.42,3.14), p<.001; Emotional never vs. yes but not in past 12 months: 1.64 (0.83,3.26), Emotional never vs. often or sometimes, 1.71 (1.13,2.60), p<.05; Sexual never vs. yes but not in past 12 months, 1.10 (0.38,3.21); Sexual never vs. often or sometimes, 1.89 (1.21,2.95), p<.01; IPV and cessation of exclusive breastfeeding: No significant relationships in Malawi or Zambia. In Tanzania, emotional ever, 1.89 (0.94,3.77); emotional never vs. yes but not in past 12 months, 0.70 (0.14,3.55), Emotional never vs. often or sometimes, 2.23 (1.09,4.57), p<.05. | Models adjusted for strata, cluster, child sex, maternal age, maternal occupation, maternal wealth index, residence (urban or rural), maternal education, maternal literacy, maternal exposure to radio, newspaper, or TV, type of delivery (C-section or vaginal), delivery location, birth spacing, and antenatal visits. | No | No | Delayed initiation: In all three countries, mothers who experienced sexual IPV were more likely to delay breastfeeding; in Malawi and Tanzania, emotional violence was associated with delayed initiation. Nonexclusive breastfeeding: Associations between IPV and EBF generally differed across countries. The only similarity was that women in both Malawi and Zambia who experienced sexual IPV had increased odds of not exclusively breastfeeding. Additionally, in Zambia, infants born to mothers who experienced physical IPV were more likely not to be exclusively breastfed. Evidence signifying associations between IPV and continued breastfeeding until the child’s first birthday were lacking and the association between maternal experience of IPV and continued breastfeeding remains unclear. |
| Aristizabal 2022 | Physical violence and Exclusive Breastfeeding  aOR 1.16 (0.79 - 1.72)  Physical violence and Breastfeed at any time  Never breastfed aOR 1.06 (0.46 - 2.44) Physical violence and Initiation of breastfeeding  After the first hour of life aOR 1.07 (0.73 - 1.26) | pregnancy planning, age, schooling,  occupation, marital status, race, and parity, partner alcohol and drugs consumption, age, schooling,  type of family and wealth  index. | No | No | There was no statistically significant relationship between IPV during pregnancy and breastfeeding found in this study |
| Islam (2017) | AOR of physical IPV only and exclusive breastfeeding: 0.13 (0.06–0.30), p<.001; AOR of sexual violence only and exclusive breastfeeding, 0.32 (0.13–0.76), p<.01; Psychological violence only and exclusive breastfeeding, 0.36 (0.19–0.68), p<.01; AORs in model with all forms of violence separately included: Physical IPV only: 0.17 (0.07–0.40), p< 0.001; Sexual violence only: 0.43 (0.18–1.06), not significant; Emotional violence only: 0.51 (0.26–1.00), p<.05 | Yes - maternal education, family monthly income, place of residence, family structure (extended vs. nuclear), maternal age during last pregnancy, pregnancy intention, parity, age of the child, number of children under 5 years of age, number of antenatal care (ANC) visits, and maternal health status. Birth-related factors such as mode of birth (caesarean = 0, vaginal = 1), place of birth (birth at home = 0, birth at medical facility = 1) and complications during childbirth (no = 0, yes = 1) were also considered. Experience of child sexual abuse, social support, post partum depression, gender of last child, | No | Yes - looked at PPD and violence exposure as interaction term | During the first 6 months following childbirth, women who experienced physical IPV had an 82% greater risk of discontinuing EBF than women who had not experienced physical IPV. Additionally, women who experienced psychological IPV were 49% less likely to exclusively breastfeed than women who had not experienced psychological IPV after childbirth. |
| Hampanda (2016) | AOR of any IPV and mixed infant feeding, 2.86 (1.68–4.87), p=.000; AOR of emotional IPV and mixed infant feeding, 1.87 (1.14–3.06), p=.013, AOR of sexual IPV and mixed infant feeding, 2.25 (1.36–3.72), p=.002; AOR of number of IPV events and mixed infant feeding, 1.17 (1.04–1.33), p=.012; AOR of frequency of IPV in past year and mixed infant feeding, 1.08 (1.02–1.14), p=.011 | Yes - maternal age, age of child, parity, education, knowledge mother to child transmission, wealth, disclosed HIV status | No | No | Women who experienced IPV from their husband had 2.9 higher adjusted odds of early mixed feeding compared to women who did not experience IPV (p<0.001). Similar to the unadjusted models, physical violence did not significantly affect the adjusted odds of early mixed feeding. However, emotional and sexual IPV were both positively associated with higher adjusted odds of early mixed feeding. |
| **Child growth** | | | | | |
| Asling-Monemi (2009) | Any violence  At birth p <0.05 adjusted mean weight and length  WAZ all children n = 3164 -1.44 (-1.39 to -1.48)*  WAZ girls n = 1539 -1.44 (-1.38 to -1.51)*  WAZ boys n = 1625 -1.42 (-1.36 to -1.49)*  HAZ all children n = 3164 -1.00 (-0.94 to -1.06)*  HAZ girls n = 1539 -0.98 (-0.90 to -1.06)*  HAZ boys n = 1625 -1.02 (-0.94 to -1.10)*  At 24 months of age p <0.05 adjusted mean weight and length  WAZ all children n = 2653 -1.77 (-1.71 to -1.82)*  WAZ girls n = 1296 -1.74 (-1.66 to -1.81)*  WAZ boys n = 1357 -1.80 (-1.72 to -1.88)*  HAZ all children n = 2653 -2.11 (-2.06 to -2.16)*  HAZ girls n = 1296 -2.06 (-1.98 to -2.13)*  HAZ boys n = 1357 -2.16 (-2.08 to -2.24)*  From 0 to 24 months of age p <0.05 adjusted yearly changes in weight and length  WAZ all children n = 2653 -0.20 (-0.17 to -0.22)*  WAZ girls n = 1296 0.18 (-0.15 to -0.22)*  WAZ boys n = 1357 -0.22 (-0.18 to -0.25)*  HAZ all children n = 2653 -0.58 (-0.56 to -0.61)*  HAZ girls n = 1296 -0.57 (-0.54 to -0.60)  HAZ boys n = 1357 -0.60 (-0.56 to -0.64)* | Adjusted mean model, Asset score (high or low), mothers educational level, parity, and religion. Adjusted yearly changes in weight and height model  Asset score (high or low), mothers educational level, parity, religion and weight-for-age Z score (WAZ) or height-for-age Z score (HAZ) at birth | No | No | Experiencing domestic violence is associated with lower weight and length for age SD scores for newborns and up to 2 years old. The growth velocity was also slower for children whose mother had experience with lifetime violence. There is an increased risk of fetal growth retardation after birth not only while in the womb for those exposed to violence, implicating that the effect of violence continues after birth. |
| Sabu (2020) | Domestic Violence and Composite index of anthropometric failure AOR 1.71 (0.78‑3.73) Domestic Violence and three anthropometric failures (stunting, wasting, and underweight)  AOR 2.35 (1.02‑5.39)* | Total number of household members, Consumption of any fruits or vegetables, Household land ownership, Toilet facility at home, Food security, Education of the mother, Maternal age at marriage, Work status, Maternal alcoholic consumption, Experience of domestic violence, Birth weight, Frequency food consumption | No | No | IPV is significantly associated with anthropometric failure after adjusting. Domestic violence is not significantly associated with CIAF after adjustment but it is before adjustment. A more targeted design for food provision is needed to reach most vulnerable populations. |
| Subramanian (2008) | Domestic violence and anemia or underweight among children 12-35 months  Any anemia OR 95% CI p =0.37 More than 1 year ago AOR 0.94 (0.81, 1.10) Once in the past year AOR 1.16 (0.91, 1.47)  More than once in the past year AOR 1.06 (0.91, 1.24) Severe anemia OR 95% CI p=0.12 More than 1 year ago AOR 1.02 (0.76, 1.35) Once in the past year AOR 0.99 (0.65, 1.52) More than once in the past year AOR 1.26 (0.97, 1.63) Wasting p<0.02 More than 1 year ago AOR 0.98 (0.86, 1.12)  Once in the past year AOR 1.06 (0.87, 1.30) More than once in the past year AOR 1.18 (1.03, 1.35)* Severe wasting p=0.69 More than 1 year ago AOR 0.86 (0.72, 1.02) Once in the past year AOR 1.03 (0.81, 1.32) More than once in the past year AOR 1.05 (0.90, 1.23) Stunting p<0.04 More than 1 year ago AOR 0.94 (0.82, 1.08) Once in the past year AOR 1.25 (1.01, 1.55)* More than once in the past year AOR 1.14 (0.99, 1.31) Severe stunting p=0.28 More than 1 year ago AOR 0.99 (0.93, 1.04) Once in the past year AOR 0.98 (0.90, 1.06) More than once in the past year AOR 0.97 (0.92, 1.03) Underweight for age p =0.21 More than 1 year ago AOR 1.05 (0.89, 1.25) Once in the past year AOR 1.03 (0.80, 1.34) More than once in the past year AOR 1.11 (0.94, 1.30) Severe underweight for age p=0.01 More than 1 year ago AOR 0.95 (0.71, 1.26) Once in the past year AOR 1.34 (0.92, 1.94) More than once in the past year AOR 1.34 (1.05, 1.70)* Low body mass index for age p =0.08 More than 1 year ago AOR 1.07 (0.87, 1.30) Once in the past year AOR 0.93 (0.68, 1.28) More than once in the past year AOR 1.21 (1.01, 1.46)* Severe low body mass index for age p=0.0006 More than 1 year ago AOR 1.09 (0.80, 1.49) Once in the past year AOR 1.65 (1.11, 2.45)* More than once in the past year AOR 1.50 (1.15, 1.95)* | Rural/urban location, age, religion, caste, education, employment, living standard, recent birth, current breastfeeding, number of children born, decision-making autonomy, and affliction with recent major illness | No | No | IPV is significantly associated with malnutrition in women and children. |
| Taft (2015) | Physical violence and LBW: AOR: 2.08, 95%CI 1.64-2.64; Any type of violence and LBW: AOR 1.46, 95%CI 1.01-2.10 | Yes - age, marital status, occupation, urban or rural residence, wealth, age at first intercourse and number of children | No | No | There were no significant differences in height or weight for age or height for weight for the children of women who had experienced violence. Equally, the results show no significant difference in infant anemia. There are also serious indicators for infant health associated with violence against Timorese women. Children of abused women are more likely to have a birth weight lower than average. Although there were no further associations for poor infant health found in other countries, such as anemia or stunting this may be because poor infant health is widespread in Timor-Leste. |
| Sobkoviak (2012) | Linear regression estimates: beta and SE, physical or emotional past year violence and weight for height z score: 0.16 (0.11), not significant. sexual violence past year and weight for height z score: 0.41 (0.19) * , p<.05; physical or emotional past year violence and weight for age z score: 0.07 (0.17) , not significant. sexual violence past year and weight for age z score: -0.31 (0.18) , p<.1, physical or emotional past year violence and height for age z score: 0.07 (0.14), not significant. sexual past year violence and height for age z score: -0.48 (0.21), p<.05. AORs and SEs: past year physical or emotional violence and wasting: 1.12 (0.44), not significant. past year sexual violence and wasting, 0.61 (0.49), not significant. past year physical or emotional violence and underweight: 0.83 (0.27), not significant. past year sexual violence and underweight, 2.57 (1.02), p<.05. past year physical or emotional violence and stunting, 0.84 (0.21), not significant; past year sexual violence and stunting, 2.23 (0.57), p<.001 | Yes - multivariate analysis adjusted for fourteen household-, maternal-, and child-level variables that may confound the relationships between maternal exposure to domestic violence and child anthropometric status. Household attributes included a measure of overall crowding (total number of household members), a direct measure of the child’s competition for resources (number of resident children less than five years), wealth quintile, urban versus rural residence, and region of residence (Greater Monrovia [reference], North Western, South Central, South Eastern A & B, North Central). Attributes of the mother included her age, schooling (none [reference], primary, secondary or higher), body mass index (BMI, in kg/m2 ), marital status (married [reference], living together, divorced/ widowed/not living together), and age at first marriage in years. Attributes of the child included his or her age (<1 year [reference], 1, 2, 3, and 4 years), gender, and birth order. The last control variable included whether or not a male sibling of the mother had died during either period of the civil conflict | No | No | Sexual violence (and not physical and emotional violence) highly associated with various nutrition and health outcomes for children; associations were most evident for nutritional indicators that relate to the overall (underweight) and long-term (stunting) nutritional status of children, which strongly predict adverse outcomes into adulthood |
| Rico (2011) | AORs IPV and child stunting: Egypt: Any IPV and child stunting: 0.95 (0.75 to 1.21); Physical only and child stunting: 1.02 (0.79 to 1.32); Sexual only and child stunting: 1.27 (0.36 to 4.46); Physical and sexual: 0.64 (0.36 to 1.12). Honduras: Any IPV and child stunting: 1.03 (0.88 to 1.22); Physical only and child stunting: 1.01 (0.82 to 1.23); Sexual only and child stunting: 0.82 (0.50 to 1.33); Physical and sexual: 1.21 (0.92 to 1.59). Kenya: Any IPV and child stunting: 1.36 (1.16 to 1.61); Physical only and child stunting: 1.36 (1.13 to 1.63); Sexual only and child stunting: 1.25 (0.79 to 1.98); Physical and sexual: 1.40 (1.07 to 1.85); Malawi: Any IPV and child stunting: 1.05 (0.92 to 1.21); physical only and child stunting: 0.99 (0.84 to 1.18); sexual only and child stunting: 1.17 (0.92 to 1.49); physical and sexual: 1.10 (0.87 to 1.38); Rwanda: Any IPV and child stunting: 1.05 (0.87 to 1.27); Physical only and child stunting: 1.07 (0.86 to 1.34); Sexual only and child stunting: 1.16 (0.72 to 1.89); Physical and sexual: 0.95 (0.69 to 1.30). AORs IPV and severe child stunting: Egypt: Any IPV and severe child stunting: 0.77 (0.57 to 1.05); Physical only: 0.85 (0.61 to 1.19); Sexual only: 0.26 (0.05 to 1.50); Physical and sexual: 0.50 (0.21 to 1.18); Honduras: Any IPV: 1.03 (0.83 to 1.27); Physical only: 0.91 (0.69 to 1.20); Sexual only: 0.63 (0.33 to 1.18); Physical or sexual: 1.46 (0.98 to 2.17). Kenya: Any IPV and severe child stunting: 1.33 (1.04 to 1.69); Physical only: 1.31 (1.01 to 1.71);  Sexual only: 1.25 (0.70 to 2.25);  Physical and sexual: 1.36 (0.96 to 1.94). Malawi: Any IPV and severe child stunting: 1.09 (0.93 to 1.27); Physical only and severe child stunting: 0.98 (0.80 to 1.19); Sexual only and severe child stunting: 1.29 (0.96 to 1.74); Physical and sexual and severe child stunting: 1.15 (0.91 to 1.47). Rwanda: Any IPV and severe child stunting: 1.04 (0.85 to 1.26); Physical only and severe child stunting: 1.09 (0.86 to 1.40); Sexual only and severe child stunting: 1.13 (0.63 to 2.05); Physical and sexual: 0.89 (0.64 to 1.23) | Yes - Maternal age, maternal education, number of living children, urban/rural residence and household socioeconomic status (as measured by a country-specific wealth index). Yes: Child-level variables: were defined as whether or not the pregnancy was intended (at the time versus later/never), duration of breast feeding (never-11 months, 12-23 months, >24 months/still breast feeding) and BCG vaccination (based on vaccination record or maternal recall). Maternal level variables: antenatal care (four or more visits), tetanus toxoid coverage (received two or more tetanus toxoid vaccinations before the birth) and skilled delivery (delivered by a doctor, nurse/midwife or auxiliary nurse/midwife) | No | No | For moderate child stunting, significant associations with IPV were only observed in Kenya, with adjusted ORs for stunting of 1.40 (95% CI 1.07 to 1.85) if the respondent was exposed to physical and sexual IPV and 1.36 (95% CI 1.16 to 1.61) if exposed to any IPV (table 4). In Kenya severe stunting showed a stronger association with physical violence, whereas in Honduras and Malawi it was associated more with physical and sexual IPV and only sexual IPV, respectively. |
| Chai 2016 | AORs: Any IPV and stunting; 1.11 (1.09–1.14); Physical IPV only and stunting: 1.11 (1.09–1.14); Sexual IPV only and stunting: 1.09 (1.05–1.13); Physical and sexual IPV: 1.10 (1.05–1.14); AORs: Any IPV and wasting: 0.94 (0.90–0.98), not significant; physical IPV only: 0.95 (0.91–0.99); sexual IPV only: 1.00 (0.94–1.07); physical and sexual IPV: 1.04 (0.96–1.11). | Yes - Adjusted for maternal age, employment status, level of education, marital status, partner’s level of education, rural/urban residence, use of contraception and wealth quintile, the number of children aged less than five years in the household and the child’s age. | No | Yes - stratified results of any IPV and stunting and any IPV and wasting by: child's age (less than 24 months/ more than 24 months); child sex; residence (rural/ urban); wealth quintile; country (low income vs. middle income) and maternal education. For stunting, compared with the values for the other children in the sample, stronger positive associations with intimate partner violence were found among the children of women who had not been educated beyond primary level (aOR: 1.09; 95% CI: 1.07–1.12), who lived in urban areas (aOR: 1.22; 95% CI: 1.17–1.28), who lived in households in the two highest asset quintiles (aOR: 1.18; 95% CI: 1.14–1.22) and who lived in a middle-income country (aOR: 1.13; 95% CI: 1.10–1.17). The odds of child wasting were lower for the sampled children who were aged at least 24 months than for their younger counterparts (aOR: 0.88; 95% CI: 0.83–0.93). | Maternal exposure to IPV substantially increases a child’s risk of stunting |
| Khan (2021) | AOR of any IPV and one form of malnutrition: 1.06 (0.96-1.18), p=.262; AOR of any IPV and multiple malnutrition outcomes: 1.06 (0.94-1.20), p=.325 | Yes - child's age, mothers' education, fathers' occupation, mothers' working status, watching television, wealth index, place of residence, birth cohort | No | No | IPV not significantly associated with one or multiple types of malnutrition in adjusted models |
| Das (2020) | AOR Model 1: Physical violence and stunting: 1.98 (1.36, 2.86); AOR Model 2: 1.92 (1.3, 2.85); AOR Model 3: 1.90 (1.28, 2.81); AOR Model 4: 1.86 (1.25, 2.77); AOR Model 5: 1.83 (1.21, 2.77) | Yes -  Model I included variables on water tap at home, private toilet, socio-economic status, mother experiencing physical violence and maternal education  Model II included variables on water tap at home, private toilet, socio-economic status, mother experiencing physical violence, maternal education, birth interval and intended pregnancy  Model III included variables on water tap at home, private toilet, socio-economic status, mother experiencing physical violence, maternal education, birth interval, intended pregnancy, 4 or more antenatal care visits and institutional delivery  Model IV included variables on water tap at home, private toilet, socio-economic status, mother experiencing physical violence, maternal education, birth interval, intended pregnancy, 4 or more antenatal care visits, institutional delivery and age-appropriate feeding  Model V included variables on water tap at home, private toilet, socio-economic status, mother experiencing physical violence, birth interval, intended pregnancy, 4 or more antenatal care visits, institutional delivery, age-appropriate feeding, parental education, religion, gender and age of the child | No | No | Maternal exposure to physical IPV substantially increased a child’s risk of stunting |
| Ferraro (2017) | Past year physical violence and weight: −148.52 (−252.95/−44.09), significant; Emotional violence and weight: −62.25 (−144.05/19.55), not significant; Sexual violence and weight: −244.78 (−483.72–5.84), significant | Adjusted for socio-demographic variables: maternal schooling, economic class, adolescent childbearing, maternal migration in first models; Same adjustments plus for maternal risk behaviours: smoking during gestation, drinking during gestation, inadequate number of prenatal care visits for the gestational age and inadequate weight gain for the gestational age considering the pre-gestational BMI in second model | Yes -  tested whether the associations could be explained by the presence of maternal risk behaviours, namely smoking, drinking, inadequate prenatal care, and inadequate weight gain. Stressful events could increase the likelihood of these behaviours, which in turn could affect foetal development. If the association disappears when these variables are included in the models it means that these variables completely explain the link between mental disorders/domestic violence and neonate outcomes, since they would be in the pathway between exposure and outcome. | No | Association between IPV and weight, could not be merely explained by neglected prenatal care, lower gestational weight gain, smoking, or drinking |
| Hasselmann (2006) | Model 1: Physical violence past 12 months and SAM: 1.14 [1.04, 1.27]; Verbal and SAM: 1.08 [0.99, 1.19]; Model 2 (alcohol and birth weight as mediators): Physical violence: 1.15 [1.05, 1.27]; 1.11 [1.01, 1.21]; Severe physical violence, Model 1: 2.80 [1.19, 6.58], Model 2: 3.22 [1.38, 7.56] | Yes - household environmental condition, socio-economic status, number of children under 5, birth interval with next eldest child, maternal education, alcohol use, birthweight | Yes - alcohol use and low birth weight included as possible mediators | No | This study showed that severe physical aggression by a partner, but not minor physical or verbal aggression, was associated with SAM. |
| Barnett 2022 | *p < 0.05. **p < 0.001 Birth n=972 Emotional IPV score  Weight-for-age z-score β (95% CI) -0.04 (-0.07, -0.02)** Length-for-age z-score  β (95% CI) -0.03 (-0.07, 0.01)   Physical IPV score  Weight-for-age z-score  β (95% CI) -0.04 (-0.07, -0.02)*  Length-for-age z-score β (95% CI) -0.04 (-0.08, -0.00)*  Sexual IPV score Weight-for-age z-score  β (95% CI) -0.05 (-0.13, 0.03) Length-for-age z-score β (95% CI) -0.10 (-0.22, 0.01)  12 months n=783 Emotional IPV score  Weight-for-age z-score  β (95% CI) -0.07 (-0.11, -0.04)**  Length-for-age z-score β (95% CI) -0.07 (-0.10, -0.04)**  Physical IPV score Weight-for-age z-score  β (95% CI) -0.03 (-0.05, -0.00)* Length-for-age z-score β (95% CI) -0.03 (-0.06, 0.01)  Sexual IPV score Weight-for-age z-score  β (95% CI) -0.08 (-0.22, 0.07)  Length-for-age z-score β (95% CI) -0.10 (-0.31, 0.10) | The weight-for-age model at birth was adjusted for recruitment site,  maternal height and child sex; length-for-age model at birth was adjusted for maternal height. Length-for-age and weight-for-age at  12 months were adjusted for recruitment site, maternal education, household income, maternal height, child sex and weight-for-age z-scores at birth. | Number of hospitalizations, maternal substance use and depression, | no | There was a relationship between maternal emotional and physical IPV and lower WFAZ at birth which was mediated by tobacco and alcohol use during pregnancy. Physical IPV and lower LFAZ at birth were found to have a relationship, this relationship was mediated by alcohol. Maternal tobacco use mediated the relationship found at 12 months, between maternal emotional and physical IPV and lower infant WFAZ, and emotional IPV and lower child LFAZ. |
| Ziaei (2014) | Exposure to physical IPV Stunted AOR 1.48* (1.23–1.79); Wasted AOR 1.12 (0.88–1.42); Underweight AOR 1.14 (0.94–1.38)  Exposure to sexual IPV Stunted AOR 1.28** (1.02–1.61); Wasted AOR 1.04 (0.79–1.37); Underweight AOR 1.03 (0.82–1.29) p<0.05 Exposure to any form of physical and/or sexual IPV Stunted AOR 1.51* (1.25–1.84); Wasted AOR 1.19 (0.93–1.51); Underweight AOR 1.18 (0.98–1.43) *p<0.01 | Woman’s educational level, husband’s educational level, woman’s height, and household wealth | No | No | Maternal exposure to lifetime physical or sexual or both types of IPV has higher odds with having a stunted child |
| Chowdhury 2021 | IPV and One form of severe malnutrition 1.06 (0.96–1.18), not significant IPV and multiple forms of severe malnutrition 1.06 (0.94–1.20), not significant | age of child, sex of child, mother’s education, father’s employment status, mother’s working status, watching television, source of water, solid waste used in cooking, mother experienced intimate partner violence [IPV], birth cohort, household socioeconomic status and rural/urban | No | No | Severe child malnutrition was found to be associated with risk factors including child age, mother's education, watching television, wealth index and birth cohort. A relationship between IPV and one or more severe forms of malnutrition was found when model was not adjusted for other socio-demographic variables, the association was not significant when the models were adjusted |
| Fonseka 2022 | Stunting and Maternal Past Year Sexual IPV aOR 1.17 (0.64, 2.14), p=0.60; in areas distal to conflict: aOR: 2.71 (1.16–6.35) Stunting and Maternal Past Year Physical IPV aOR 0.95 (0.67, 1.34), p=0.76  Stunting and Maternal Past Year Emotional IPV aOR 1.04 (0.76, 1.42), p=0.80; in areas proximal to conflict: aOR: 1.80(1.13–2.89) | age in months, birth order, maternal height, maternal age, maternal education, household wealth quintile, ethnicity, and district | No | Proximity to conflict was evaluated as a moderator for a relationship between maternal child marriage and IPV and stunting | Distance to conflict impacted stunting where children closer to conflict were less stunted. Distance to conflict also moderated the impact of sexual and emotional IPV on stunting. Maternal sexual IPV increased odds of stunting when far from conflict and emotional IPV increased odds when central to conflict. The women in proximal districts to conflict showed a decreased odds in stunting for their children when exposed to emotional IPV |
| Issah 2022 | IPV and Stunting  AOR 0.94 (95% CI) (0.68, 1.30) p=0.703  IPV and Underweight AOR 0.69 (95% CI)(0.50, 0.96)* p=0.025 IPV and Wasting AOR 0.83 (95% CI) (0.52, 1.35), p= 0.467 | Listed as "child and maternal-related factors" maternal overweight status | No | No | Women exposed to IPV were more likely to be overweight and their children less likely to be underweight. |
| UysalYalcin 2022 | Physical Violence and child with Wasting and moderate wasting B -1.624 p= 0.042 Wald 4.187 Times 5.07 df 1 ExpB 0.197 Emotional Violence during pregnancy and child with Wasting and moderate wasting B -2.035 p=0.017 Wald 5.698 Times 7.63 df 1 ExpB 0.131 Emotional Violence and child with Wasting and moderate wasting B 1.942 p=0.029 Wald 4.769 Times 0.14 df 1 ExpB 6.976 Emotional Violence during pregnancy and Moderate overweight and overweight B 1.647 p=0.035 Wald 4.433 Times 0.19 df 1 ExpB 5.19 Controlling behaviour during pregnancy and child with moderate overweight and overweight B -1.600 p=0.032 Wald 4.582 Times 4.95 df 1 ExpB 0.20 Physical Violence during pregnancy and child with moderate tall and tall height B 1.955 p=0.022 Wald 5.280 Times 0.14 df 1 ExpB 7.065 Controlling behaviour and child with moderate tall and tall height B 1.447 p=0.044 Wald 4.063 Times 0.23 df 1 ExpB 4.248 | None listed | No | No | Child growth is associated with maternal exposure to IPV. |
| Neamah (2018) | Sexual IPV and Stunting (n = 908) SMD 1.5** (1.21 to 1.94) p<0.01 Physical IPV and stunting SMD 1.12 (0.90 to 1.12)  Physical and/ or sexual IPV and stunting SMD 1.59** (1.20 to 2.23) p<0.01 | Age, maternal education, child's age, Bayley assessor, Vitamin A random assignment and wealth quintiles | No | No | Maternal exposure to IPV and maternal depression is associated with stunting (nutritional) outcomes and developmental outcomes. |
| Salazar (2012) | IPV exposure Adjusted mean height-for-age Z-scores Boys n = 178 Girls n = 197 Total n = 375  Any IPV during pregnancy Yes Boys −1.03 (−1.20 to −0.87); Girls −1.14 (−1.32 to −0.96) Total −1.09 (−1.21 to −0.98) No Boys−0.96 (−1.18 to −0.73) −0.76 (−0.98 to −0.54)*; Total −0.85 (−1.01 to −0.69)*  Emotional IPV during pregnancy Boys Yes −0.92 (−1.15 to −0.68) ;Girls −1.05 (−1.31 to −0.79); Total −0.99(−1.16 to −0.81) No Boys−1.05 (−1.21 to −0.89); Girls −0.97 (−1.14 to −0.80); Total −1.01 (−1.12 to −0.89)  Physical IPV during pregnancy Boys Yes −1.07 (−1.43 to −0.71); Girls −1.20 (−1.58 to −0.81); Total −1.13 (−1.40 to −0.87) No Boys−1.00 (−1.14 to −0.85); Girls −0.96 (−1.11 to −0.81); Total −0.98 (−1.08 to −0.88)  Sexual IPV during pregnancy Boys Yes −1.02 (−1.53 to −0.51); Girls −1.04 (−1.63 to −0.44); Total −1.03 (−1.42 to −0.64) No Boys−1.01 (−1.15 to −0.86); Girls −0.99 (−1.14 to −0.84); Total −1.00 (−1.10 to −0.89)  Controlling behavior by maternal partner Boys Yes −1.03 (−1.20 to −0.85); Girls −1.23 (−1.42 to −1.03); Total −1.13 (−1.26 to −1.00) No Boys−0.98 (−1.19 to −0.77); Girls −0.74 (−0.94 to −0.54)*; Total −0.84 (−0.99 to −0.70)* Final model: significantly lower mean height for age z scores in girls (not boys) (0.49 zscore lower) whose mothers were affected by controlling behavior of father during pregnancy. | Any IPV during pregnancy, Emotional IPV during pregnancy and Controlling behavior by partner adjusted for residency (urban rural), mother's education, child age. Physical IPV during pregnancy adjusted for mothers education and residency.  Sexual IPV during pregnancy adjusted for parity. | No | Yes - Sex of child | Exposure to controlling behavior IPV by father of the child shows an association with lower height for age mean z-scores. When stratified, significant only for girls |
| Rahman (2012) | Form of IPV Any physical or sexual IPV or both Stunting AOR 1.59 (1.23, 2.08)*; Underweight AOR1.33 (1.04, 1.71)*; Wasting AOR 1.08 (0.78, 1.49) Physical IPV only Stunting AOR 1.40 (1.02, 1.94)*; Underweight AOR 1.23 (0.90, 1.69); Wasting AOR 1.01 (0.70, 1.47) Sexual IPV only Stunting AOR 1.64 (1.00, 2.67); Underweight AOR 1.33 (0.77, 2.27); Wasting AOR 1.07 (0.56, 2.02) Both physical and sexual IPV Stunting AOR 2.07 (1.24, 3.46)*; Underweight AOR 1.60 (1.02, 2.51)*; Wasting AOR 1.23 (0.68, 2.25) No. of types of physical IPV 1 Stunting AOR 1.48 (0.88, 2.46); Underweight AOR 1.02 (0.62, 1.67); Wasting AOR 0.87 (0.49, 1.53) 2 Stunting AOR 1.50 (0.99, 2.28);Underweight AOR 1.42 (0.96, 2.11); Wasting AOR 1.21 (0.64, 2.26) ‡3 Stunting AOR 1.57 (1.00, 2.46)* marginally sig ; Underweight AOR 1.49 (0.97, 2.30); Wasting AOR 1.19 (0.74, 1.90) | Maternal age, maternal education, maternal decision-making autonomy, maternal occupation, maternal body mass index, parity, residence, number of household members, child gender, child age, initiation of breastfeeding, duration of breastfeeding, recent child illness, and wealth index category. | No | No | Physical and sexual IPV associated with stunting and underweight in children under 5. Preventing IPV physical and sexual is important in affecting child nutritional outcomes in Bangladesh. Proportion of stunted children increases at 2 years, this may be because they have to feed and fend more for themselves at this age. |
| **Nutrient blood markers** | | | | | |
| Ziaei (2019) | Before Childbirth (BCB), After Childbirth (ACB), Both before and after Child Birth (BACB), High Density Lipoprotein (HDL), Low Density Lipoprotein (LDL) Maternal Experience of Any Lifetime Domestic Violence Apo A (g/l) Adjusted BCB −0.04 (−0.08, −0.01) *, ACB −0.02 (−0.05, 0.01), BACB −0.03 (−0.06, 0.00) P<0.05 Apo B (g/l) Adjusted BCB 0.00 (−0.03, 0.03); ACB −0.02 (−0.05, 0.01); BACB −0.00 (−0.03, 0.02) Apo B/Apo A Adjusted BCB 0.02 (−0.02, 0.05); ACB −0.01 (−0.04, 0.02); BACB 0.01 (−0.02, 0.03) HDL (mmol/l) Adjusted BCB −0.04 (−0.09, 0.00); ACB −0.03 (−0.07, 0.01); BACB −0.03 (−0.06, 0.01) LDL (mmol/l) Adjusted BCB 0.01 (−0.13, 0.14); ACB 0.01 (−0.11, 0.13); BACB 0.04 (−0.07, 0.15) LDL/HDL Adjusted BCB 0.09 (−0.06, 0.23); ACB 0.04 (−0.09, 0.17); BACB 0.10 (−0.02, 0.21) Cholesterol (mmol/l) Adjusted BCB −0.05 (−0.20, 0.09); ACB −0.08 (−0.20, 0.04); BACB −0.02 (−0.14, 0.09) Triglycerides 2 (mmol/l) Adjusted BCB 0.03 (−0.04, 0.10); ACB −0.02 (−0.08, 0.04); BACB 0.01 (−0.05, 0.07) Maternal Experience of Any Physical Domestic Violence Apo A (g/l) Adjusted BCB −0.02 (−0.07, 0.02); ACB −0.02 (−0.04, 0.01); BACB −0.02 (−0.05, 0.01) Apo B (g/l) Adjusted BCB 0.01 (−0.03, 0.05); ACB −0.01 (−0.04, 0.01); BACB 0.00 (−0.03, 0.03) Apo B/Apo A Adjusted BCB 0.02 (−0.02, 0.06); ACB −0.00 (−0.03, 0.02); BACB 0.01 (−0.02, 0.03) HDL (mmol/l) Adjusted BCB −0.03 (−0.08, 0.03); ACB −0.02 (−0.05, 0.01); BACB −0.02 (−0.06, 0.02) LDL (mmol/l) Adjusted BCB 0.05 (−0.12, 0.21); ACB 0.05 (−0.05, 0.15); BACB 0.07 (−0.05, 0.19) LDL/HDL Adjusted BCB 0.10 (−0.08, 0.28); ACB 0.09 (−0.02, 0.19); BACB 0.12 (−0.01, 0.25) Cholesterol (mmol/l) Adjusted BCB 0.00 (−0.17, 0.18); ACB −0.04 (−0.14, 0.06); BACB 0.03 (−0.10, 0.15) Triglycerides 2 (mmol/l) Adjusted BCB −0.02 (−0.11, 0.07); ACB 0.01 (−0.04, 0.06); BACB 0.07 (0.01, 0.14) * P<0.05 Maternal Experience of Any Sexual Domestic Violence Apo A (g/l) Adjusted BCB −0.05 (−0.08, −0.01); ACB ** −0.00 (−0.03, 0.02); BACB −0.02 (−0.05, 0.02) P<0.01 Apo B (g/l) Adjusted BCB 0.03 (0.00, 0.06); ACB * −0.01 (−0.03, 0.02); BACB 0.02 (−0.01, 0.04) P<0.05 Apo B/Apo A Adjusted BCB 0.04 (0.02, 0.07); ACB −0.00 (−0.02, 0.02); BACB 0.02 (−0.01, 0.05) HDL (mmol/l) Adjusted BCB −0.05 (−0.10, −0.01); ACB ** −0.00 (−0.03, 0.03); BACB −0.00 (−0.04, 0.04) P<0.01 LDL (mmol/l) Adjusted BCB 0.17 (0.05, 0.29); ACB ** −0.02 (−0.12, 0.07); BACB 0.04 (−0.08, 0.16) P<0.01 LDL/HDL Adjusted BCB 0.24 (0.11, 0.38); ACB ** −0.01 (−0.11, 0.10); BACB 0.04 (−0.09, 0.17) P<0.01 Cholesterol (mmol/l) Adjusted BCB 0.05 (−0.08, 0.17); ACB −0.03 (−0.14, 0.07); BACB 0.08 (−0.04, 0.20) Triglycerides 2 (mmol/l) Adjusted BCB 0.06 (−0.01, 0.12); ACB −0.02 (−0.07, 0.03); BACB −0.01 (−0.07, 0.05)  Maternal Experience of Any Controlling Behavior Apo A (g/l) Adjusted 1 Ref −0.02 (−0.04, 0.01) −0.01 (−0.04, 0.02); BACB −0.03 (−0.06, −0.00) * P<0.05 Apo B (g/l) Adjusted 1 Ref −0.01 (−0.03, 0.02) −0.02 (−0.05, 0.00); BACB 0.00 (−0.02, 0.03) Apo B/Apo A Adjusted 1 Ref 0.01 (−0.02, 0.03) −0.02 (−0.04, 0.01); BACB 0.01 (−0.01, 0.04) HDL (mmol/l) Adjusted 1 Ref −0.01 (−0.04, 0.03) −0.02 (−0.06, 0.02); BACB −0.03 (−0.07, 0.00) LDL (mmol/l) Adjusted 1 Ref −0.05 (−0.15, 0.06) −0.17 (−0.28, −0.06) **; BACB 0.01 (−0.10, 0.12) P<0.01 LDL/HDL Adjusted 1 Ref −0.02 (−0.14, 0.09) −0.12 (−0.25, −0.00) *; BACB 0.08 (−0.04, 0.20) P<0.05 Cholesterol (mmol/l) Adjusted 1 Ref −0.05 (−0.16, 0.06) −0.13 (−0.25, −0.02) *; BACB −0.03 (−0.15, 0.08) P<0.05 Triglycerides 2 (mmol/l) Adjusted 1 Ref −0.00 (−0.06, 0.05) −0.01 (−0.07, 0.05); BACB 0.01 (−0.04, 0.07) | Maternal Experience of Any Lifetime DV maternal education, SES and age Maternal Experience of Any Physical Domestic Violence maternal education, SES and age; pregnancy supplementation and feeding intervention group (randomized trial participants) | No | No | Direction and strength of association varied by type of violence. Maternal physical violence before and after birth and sexual domestic violence before birth may negatively affect children's lipid profile at 10yrs. The lipid profile for children of women who experienced controlling behavior after birth were better than the comparing group. Children of women who experienced any domestic violence had more unfavorable lipid profiles. (56% of women experienced DV prenatally, while 66% experienced DV postnatally); A large proportion of children were stunted (28%) or underweight (48.5%) but this was not accounted for in the analyses of the relationship of exposure to DV and child lipid levels. |
| Avci 2022 | Domestic Violence and LBW x2= 0.043, p=0.836 Domestic violence during pregnancy and Breastfeeding self efficacy z=-3.309*, p<0.05 Domestic Violence and Cortisol levels in newborns Z=-2.922*, p=0.003 Physical Violence and Cortisol levels in newborns Z=-0.839, p=0.402 Emotional Violence and Cortisol levels in newborns Z=-2.818*, p = 0.005 Sexual Violence and Cortisol levels in newborns Z=-2.087*, p = 0.037 Economic Violence and Cortisol levels in newborns Z=-1.431, p = 0.153 | None listed | No | No | Domestic violence during pregnancy shows higher cortisol hormone levels in newborns than in those not exposed to DV. DV during pregnancy affects breastfeeding after delivery. |
