# Supplementary material for: Linkages between maternal experience of intimate partner violence and child nutrition outcomes: A rapid evidence assessment
Source: PLoS One. 2024 Mar 18;19(3):e0298364. doi: 10.1371/journal.pone.0298364 (PMC10947923; doi:10.1371/journal.pone.0298364)
Supplement: S2 Table — (DOCX) [file pone.0298364.s003.docx]

S2 Table: Quality assessment

| **Article** | **Was the research question or objective in this paper clearly stated?** | **Representativeness of the sample (external validity)*** | **Was the target/reference population clearly defined? (Is it clear who the research was about?) (internal validity)** | **Is the sampling strategy relevant to address the research question? (internal validity)** | **Non-Respondents (internal validity)** | **Is the violence exposure measured rigorously? (internal validity)** | **Is the nutrition outcome measured rigorously? (internal validity)** | **Were key potential confounding variables measured and adjusted for?** | **Statistical test***** |
| --- | --- | --- | --- | --- | --- | --- | --- | --- | --- |
| Frith (2017) | Yes | Selected group (i.e. clinic-based sampling in hospital) | No | Yes | Satisfactory response rate or comparability | Yes | Yes | Yes | Appropriate statistical test |
| Khan (2020) | Yes | Representative sample | Yes | Yes | No response rate or comparability included | Cannot determine | Yes | Yes | Appropriate statistical test |
| Neamah (2018) | Yes | Representative sample | No | Yes | Satisfactory response rate or comparability | Yes | Yes | Yes | Appropriate statistical test |
| Vo (2019) | Yes | Representative sample | Yes | Yes | Satisfactory response rate or comparability | Yes | Yes | Yes | Appropriate statistical test |
| Tran (2020) | Yes | Representative sample | No | Yes | No response rate or comparability included | Yes | Yes | Yes | Appropriate statistical test |
| Madsen (2019) | Yes | No description of the sampling strategy | Yes | Cannot determine | Satisfactory response rate or comparability | Yes | Yes | Yes | Appropriate statistical test |
| Salazar (2012) | Yes | Representative sample | No | Yes | Satisfactory response rate or comparability | Yes | Yes | Yes | Appropriate statistical test |
| Caleyachetty (2019) | Yes | Representative sample | Yes | Yes | Satisfactory response rate or comparability | Yes | Yes | Yes | Appropriate statistical test |
| Hoang (2016) | Yes | Representative sample | Yes | Yes | No response rate or comparability included | Yes | Yes | Yes | Appropriate statistical test |
| Kana (2020) |  | Selected group (i.e. clinic-based sampling in hospital) | Yes | Cannot determine | No response rate or comparability included | Yes | Yes | Yes | Appropriate statistical test |
| Rahman (2012) | Yes | Representative sample | No | Yes | No response rate or comparability included | Yes | Yes | Yes | Appropriate statistical test |
| Ariyo (2021) | Yes | Representative sample | No | Yes | Satisfactory response rate or comparability | Yes | Yes | Yes | Appropriate statistical test |
| Ziaei (2014) | Yes | Representative sample | Yes | Yes | Satisfactory response rate or comparability | Yes | Yes | Yes | Appropriate statistical test |
| Ziaei (2019) | Yes | Representative sample | Yes | Yes | Satisfactory response rate or comparability | Yes | Yes | Yes | Appropriate statistical test |
| Ferdos (2017) | Yes | Selected group (i.e. clinic-based sampling in hospital) | Yes | Yes | Satisfactory response rate or comparability | Yes | Yes | Yes | Appropriate statistical test |
| Batool (2018) | Yes | Representative sample | Yes | Yes | No response rate or comparability included | Yes | Yes | Yes | Appropriate statistical test |
| Asling-Monemi (2009) | Yes | Representative sample | Yes | Yes | No response rate or comparability included | Yes | Yes | Yes | Appropriate statistical test |
| Valladares (2002) | Yes | Selected group (i.e. clinic-based sampling in hospital) | Yes | Yes | No response rate or comparability included | Yes | Yes | Yes | Appropriate statistical test |
| Valladares (2009) | Yes | Somewhat representative of the average in the target population. (non-random sampling) | Yes | Yes | No response rate or comparability included | Yes | Yes | Yes | Appropriate statistical test |
| Rahman (2021) | Yes | Representative sample | Yes | Yes | No response rate or comparability included | Yes | Yes | Yes | Appropriate statistical test |
| Marimuthu (2019) | Yes | Selected group (i.e. clinic-based sampling in hospital) | Yes | Yes | No response rate or comparability included | Yes | Yes | Yes | Appropriate statistical test |
| Tiwari (2018) | Yes | Representative sample | Yes | Yes | No response rate or comparability included | Yes | Yes | Yes | Appropriate statistical test |
| Sabu (2020) | Yes | Representative sample | Yes | Yes | No response rate or comparability included | Yes | Yes | Yes | Appropriate statistical test |
| Subramanian (2008) | Yes | Representative sample | Yes | Yes | Satisfactory response rate or comparability | Yes | Yes | Yes | Appropriate statistical test |
| Boyce (2017) | Yes | Representative sample | Yes | Yes | No response rate or comparability included | Yes | Yes | Yes | Appropriate statistical test |
| Young (2020) | Yes | Representative sample | Yes | Yes | No response rate or comparability included | Yes | Yes | Yes | Appropriate statistical test |
| Zureick-Brown (2015) | Yes | Representative sample | Yes | Yes | No response rate or comparability included | Yes | Yes | Yes | Appropriate statistical test |
| Misch (2014) | Yes | Representative sample | Yes | Yes | Satisfactory response rate or comparability | Yes | Yes | Yes | Appropriate statistical test |
| Kaye (2006) | Yes | Selected group (i.e. clinic-based sampling in hospital) | Yes | Yes | Satisfactory response rate or comparability | Yes | Yes | Yes | Appropriate statistical test |
| Sigalla (2017) | Yes | Selected group (i.e. clinic-based sampling in hospital) | Yes | Yes | Satisfactory response rate or comparability | Yes | Yes | Yes | Appropriate statistical test |
| Musa (2021) | Yes | Selected group (i.e. clinic-based sampling in hospital) | Yes | Yes | Satisfactory response rate or comparability | Yes | Yes | Yes | Appropriate statistical test |
| Tsedal (2021) | Yes | Representative sample | Yes | Cannot determine | No response rate or comparability included | Yes | Yes | Yes | Appropriate statistical test |
| Walters (2021) | Yes | Representative sample | Yes | Yes | Satisfactory response rate or comparability | Yes | Yes | Yes | Appropriate statistical test |
| Shamu (2018) | Yes | Selected group (i.e. clinic-based sampling in hospital) | Yes | Yes | Satisfactory response rate or comparability | Yes | Yes | Yes | Appropriate statistical test |
| Laelago (2017) | Yes | Selected group (i.e. clinic-based sampling in hospital) | Yes | Cannot determine | Satisfactory response rate or comparability | Yes | Yes | Yes | Appropriate statistical test |
| Taft (2015) | Yes | Representative sample | Yes | Yes | Satisfactory response rate or comparability | Yes | Yes | Yes | Appropriate statistical test |
| Jaraba (2019) | Yes | Representative sample | Yes | Yes | Satisfactory response rate or comparability | Yes | Yes | Yes | Appropriate statistical test |
| Assefa (2012) | Yes | Not clear | Yes | Cannot determine | No response rate or comparability included | Yes | Yes | Yes | Appropriate statistical test |
| Alemu (2019) | Yes | Selected group (i.e. clinic-based sampling in hospital) | Yes | Yes | Satisfactory response rate or comparability | Cannot determine | Cannot determine | Yes | Appropriate statistical test |
| Sobkoviak (2012) | Yes | Representative sample | Yes | Yes | Satisfactory response rate or comparability | Yes | Yes | Yes | Appropriate statistical test |
| Berhanie (2019) | Yes | Selected group (i.e. clinic-based sampling in hospital) | Yes | Yes | Satisfactory response rate or comparability | Yes | No | Yes | Appropriate statistical test |
| Rico (2011) | Yes | Representative sample | Yes | Yes | Satisfactory response rate or comparability | Yes | Yes | Yes | Appropriate statistical test |
| Chai (2016) | Yes | Representative sample | Yes | Yes | Satisfactory response rate or comparability | Yes | Yes | Yes | Appropriate statistical test |
| Abujilban (2017) | Yes | Selected group (i.e. clinic-based sampling in hospital) | Yes | Yes | Response rate or comparability is unsatisfactory | Yes | Yes | No | Appropriate statistical test |
| Khan (2021) | Yes | Representative sample | Yes | Yes | No response rate or comparability included | Cannot determine | Yes | Yes | Appropriate statistical test |
| Eno (2014) | Yes | Selected group (i.e. clinic-based sampling in hospital) | Yes | Yes | Satisfactory response rate or comparability | Yes | Cannot determine | N/A | Appropriate statistical test |
| Hampanda (2016) | Yes | Selected group (i.e. clinic-based sampling in hospital) | Yes | Yes | No response rate or comparability included | Yes | Yes | Yes | Appropriate statistical test |
| Islam (2017) | Yes | Selected group (i.e. clinic-based sampling in hospital) | Yes | Yes | Satisfactory response rate or comparability | Yes | Yes | Yes | Appropriate statistical test |
| Pun (2019) | Yes | Selected group (i.e. clinic-based sampling in hospital) | Yes | Yes | Response rate or comparability is unsatisfactory | Yes | Yes | Yes | Appropriate statistical test |
| Das (2020) | Yes | Somewhat representative of the average in the target population. (non-random sampling) | Yes | Yes | Satisfactory response rate or comparability | Cannot determine | Yes | Yes | Appropriate statistical test |
| Mezmur (2021) | Yes | Selected group (i.e. clinic-based sampling in hospital) | Yes | No | No response rate or comparability included | No | Yes | Yes | Appropriate statistical test |
| Arcos (2001) | Yes | Selected group (i.e. clinic-based sampling in hospital) | Yes | Yes | No response rate or comparability included | Yes | Yes | No | The statistical test is not appropriate not described or incomplete. |
| Arcos (2003) | Yes | Selected group (i.e. clinic-based sampling in hospital) | Yes | Yes | No response rate or comparability included | Yes | Yes | No | Appropriate statistical test |
| Ruiz Grosso (2014) | Yes | Representative sample | Yes | Yes | Satisfactory response rate or comparability | Yes | Yes | Yes | Appropriate statistical test |
| Faramarzi (2005) | Yes | Selected group (i.e. clinic-based sampling in hospital) | No | Yes | No response rate or comparability included | Yes | Yes | Yes | Appropriate statistical test |
| Ferraro (2017) | Yes | Selected group (i.e. clinic-based sampling in hospital) | Yes | Yes | Response rate or comparability is unsatisfactory | Yes | Yes | Yes | Appropriate statistical test |
| Hasselmann (2016) | Yes | Selected group (i.e. clinic-based sampling in hospital) | Yes | No | Response rate or comparability is unsatisfactory | Yes | Yes | Yes | Appropriate statistical test |
| Dolatian (2016) | Yes | Selected group (i.e. clinic-based sampling in hospital) | Yes | Yes | Satisfactory response rate or comparability | Yes | Cannot determine | Yes | Appropriate statistical test |
| Lobato (2018) | Yes | Selected group (i.e. clinic-based sampling in hospital) | Yes | Yes | Satisfactory response rate or comparability | Yes | Yes | Yes | Appropriate statistical test |
| Mahmoodi (2019) | Yes | Selected group (i.e. clinic-based sampling in hospital) | Yes | Yes | Response rate or comparability is unsatisfactory | Yes | Cannot determine | Yes | Appropriate statistical test |
| Nojomi (2006) | Yes | Selected group (i.e. clinic-based sampling in hospital) | Yes | No | No response rate or comparability included | Yes | Cannot determine | Cannot determine | Appropriate statistical test |
| Hasselmann (2006) | Yes | Selected group (i.e. clinic-based sampling in hospital) | Yes | Yes | Satisfactory response rate or comparability | Yes | Yes | Yes | Appropriate statistical test |
| Mezzavilla (2016) | Yes | Selected group (i.e. clinic-based sampling in hospital) | Yes | Yes | No response rate or comparability included | Yes | Yes | Yes | Appropriate statistical test |
| Nejatizade (2017) | Yes | Selected group (i.e. clinic-based sampling in hospital) | Yes | Yes | No response rate or comparability included | Yes | Cannot determine | Cannot determine | Appropriate statistical test |
| Nunes (2011) | Yes | Selected group (i.e. clinic-based sampling in hospital) | Yes | No | No response rate or comparability included | Yes | Cannot determine | Yes | Appropriate statistical test |
| Nasreen (2019) | Yes | Selected group (i.e. clinic-based sampling in hospital) | Yes | No | Response rate or comparability is unsatisfactory | Yes | Yes | Yes | Appropriate statistical test |
| Moraes (2011) | Yes | Selected group (i.e. clinic-based sampling in hospital) | Yes | Yes | Satisfactory response rate or comparability | Yes | Yes | Yes | Appropriate statistical test |
| Ribeiro (2021) | Yes | Selected group (i.e. clinic-based sampling in hospital) | Yes | No | Response rate or comparability is unsatisfactory | Yes | Yes | Cannot determine | Appropriate statistical test |
| Caprara (2020) | Yes | Selected group (i.e. clinic-based sampling in hospital) | Yes | No | No response rate or comparability included | Yes | Yes | Yes | Appropriate statistical test |
| Abdollahi (2015) | Yes | Selected group (i.e. clinic-based sampling in hospital) | Yes | Yes | Satisfactory response rate or comparability | Yes | Yes | Yes | Appropriate statistical test |
| Khodakarami (2009) | Yes | Selected group (i.e. clinic-based sampling in hospital) | Yes | No | Response rate or comparability is unsatisfactory | Yes | Yes | Cannot determine | The statistical test is not appropriate not described or incomplete. |
| Abadi (2013) | Yes | Selected group (i.e. clinic-based sampling in hospital) | No | No | No response rate or comparability included | Cannot determine | Cannot determine | Yes | Appropriate statistical test |
| Aristizabal 2022 |  | Representative sample | Yes | Yes | No response rate or comparability included | Yes | Yes | Yes | Appropriate statistical test |
| Barnett 2022 | Yes | Selected group (i.e. clinic-based sampling in hospital) | Yes | Yes | No response rate or comparability included | Yes | Yes | Yes | Appropriate statistical test |
| Chandra 2021 | Yes | Selected group (i.e. clinic-based sampling in hospital) | Yes | Yes | No response rate or comparability included | Yes | Yes | Yes | Appropriate statistical test |
| Avci 2022 | Yes | Selected group (i.e. clinic-based sampling in hospital) | No | Yes | No response rate or comparability included | Yes | Yes | Cannot determine | Appropriate statistical test |
| Chowdhury 2021 | Yes | Representative sample | Yes | Yes | No response rate or comparability included | Yes | Yes | Yes | Appropriate statistical test |
| Debele 2022 | Yes | Selected group (i.e. clinic-based sampling in hospital) | Yes | Yes | No response rate or comparability included | Yes | Yes | Yes | Appropriate statistical test |
| Doke 2021 | Yes | Somewhat representative of the average in the target population. (non-random sampling) | Yes | Yes | No response rate or comparability included | Cannot determine | Yes | Yes | Appropriate statistical test |
| Fonseka 2022 | Yes | Representative sample | Yes | Yes | No response rate or comparability included | Yes | Yes | Yes | Appropriate statistical test |
| Issah 2022 | Yes | Representative sample | Yes | Yes | No response rate or comparability included | Yes | Yes | Yes | Appropriate statistical test |
| Woldetensay 2021 | Yes | Somewhat representative of the average in the target population. (non-random sampling) | Yes | Yes | No response rate or comparability included | Yes | Yes | Cannot determine | Appropriate statistical test |
| Okunola 2021 | Yes | Selected group (i.e. clinic-based sampling in hospital) | Yes | Yes | No response rate or comparability included | Yes | Yes | Yes | Appropriate statistical test |
| Tesfa 2021 | Yes | Selected group (i.e. clinic-based sampling in hospital) | Yes | Yes | No response rate or comparability included | Yes | Yes | Cannot determine | Appropriate statistical test |
| UysalYalcin 2022 | Yes | Selected group (i.e. clinic-based sampling in hospital) | Yes | Cannot determine | No response rate or comparability included | Yes | Yes | Cannot determine | Appropriate statistical test |
| Vachhani 2022 | No | Selected group (i.e. clinic-based sampling in hospital) | Yes | Cannot determine | No response rate or comparability included | Cannot determine | Cannot determine | No | The statistical test is not appropriate not described or incomplete. |

*1) Truly representative of the average in the target population (all subjects or random sampling), 2) Somewhat representative of the average in the target population (non-random sampling), 3) Selected group (i.e. clinic-based, sampling in hospital), 4) No description of the sampling strategy

** 1) Comparability between respondents and non-respondents’ characteristics is established, and the response rate is satisfactory, 2) The response rate is unsatisfactory, or the comparability between respondents and non-respondents is unsatisfactory, 3) No description of the response rate or the characteristics of the responders and the non-responders.

***1) The statistical test used to analyse the data is clearly described and appropriate, and the measurement of the association is presented, including confidence intervals and the probability level (p-value), and 2) The statistical test is not appropriate, not described or incomplete.
