# Supplementary material for: Linkages between maternal experience of intimate partner violence and child nutrition outcomes: A rapid evidence assessment
Source: PLoS One. 2024 Mar 18;19(3):e0298364. doi: 10.1371/journal.pone.0298364 (PMC10947923; doi:10.1371/journal.pone.0298364)
Supplement: Attachment — Submitted filename: Comment_Suggestion.docx [file pone.0298364.s006.docx]

**Comment 1.**

Line Number: 169 – 184 (PECO framework): convert into tabulation form.

**Comment 2.**

Line Number 195 -197: This line will start after the sentences of data extraction. Start from data extraction sentences after completion then author writes the quality assessment sentences.
