# Supplementary material for: Linkages between maternal experience of intimate partner violence and child nutrition outcomes: A rapid evidence assessment
Source: PLoS One. 2024 Mar 18;19(3):e0298364. doi: 10.1371/journal.pone.0298364 (PMC10947923; doi:10.1371/journal.pone.0298364)
Supplement: Attachment — Submitted filename: Response to reviewers.docx [file pone.0298364.s007.docx]

**Response to reviewers:**

Comment 1.
Line Number: 169 – 184 (PECO framework): convert into tabulation form.

We have converted this to a table.

Comment 2.
Line Number 195 -197: This line will start after the sentences of data extraction. Start from data extraction sentences after completion then author writes the quality assessment sentences.

We have edited as suggested.
