# Supplementary material for: Linkages between maternal experience of intimate partner violence and child nutrition outcomes: A rapid evidence assessment
Source: PLoS One. 2024 Mar 18;19(3):e0298364. doi: 10.1371/journal.pone.0298364 (PMC10947923; doi:10.1371/journal.pone.0298364)
Supplement: Attachment — Submitted filename: Reviewer comments.docx [file pone.0298364.s004.docx]

At the outset, I would like to congratulate the team to work on the relevant topic of IPV of maternal caregivers and child nutrition outcomes which though have been researched several times, needs periodic re-examination. Moreover, the approach to the study i.e., applying REA makes the study unique. It is an extremely well written & well researched article. Although I have a few minor queries, I would be happy to see it published.

1. Define LMIC.
2. Why to include adolescents?
3. Please elaborate line number 159-160
4. Methodology adopted to plot Harvest plot should be explained. Perhaps, after data synthesis (line no. 191- 194), the authors can add a section on the methodology adopted in the article.
